# Measurement properties of the EQ-5D in populations with a mean age of ≥ 75 years: a systematic review

**DOI:** 10.1007/s11136-022-03185-0

**Published:** 2022-08-01

**Authors:** Sophie Gottschalk, Hans-Helmut König, Mona Nejad, Judith Dams

**Affiliations:** Department of Health Economics and Health Services Research, University Medical Center Hamburg-Eppendorf, Martinistraße 52, 20246 Hamburg, Germany

**Keywords:** EQ-5D, Older population, Oldest-old population, Psychometric properties, Systematic review

## Abstract

**Purpose:**

Healthcare interventions for middle-old and oldest-old individuals are often (economically) evaluated using the EQ-5D to measure health-related quality of life (HrQoL). This requires sufficient measurement properties of the EQ-5D. Therefore, the current study aimed to systematically review studies assessing the measurement properties of the EQ-5D in this population.

**Methods:**

The databases PubMed, Cochrane library, Web of Science, Embase, and EconLit were searched for studies providing empirical evidence of reliability, validity, and/or responsiveness of the EQ-5D-3L and EQ-5D-5L in samples with a mean age ≥ 75 years. Studies were selected by two independent reviewers, and the methodological quality was assessed using the COSMIN Risk of Bias checklist. Results were rated against updated criteria for good measurement properties (sufficient, insufficient, inconsistent, indeterminate). The evidence was summarized, and the quality of evidence was graded using a modified GRADE approach.

**Results:**

For both EQ-5D versions, high-quality evidence for sufficient convergent validity was found. Known-groups validity was sufficient for the EQ-5D-5L (high-quality evidence), whereas the results were inconsistent for the EQ-5D-3L. Results regarding the reliability were inconsistent (EQ-5D-3L) or entirely lacking (EQ-5D-5L). Responsiveness based on correlations of change scores with instruments measuring related/similar constructs was insufficient for the EQ-5D-3L (high-quality evidence). For the EQ-5D-5L, the available evidence on responsiveness to change in (Hr)QoL instruments was limited.

**Conclusion:**

Since the responsiveness of the EQ-5D in a population of middle-old and oldest-old individuals was questionable, either using additional instruments or considering the use of an alternative, more comprehensive instrument of (Hr)QoL might be advisable, especially for economic evaluations.

**Supplementary Information:**

The online version contains supplementary material available at 10.1007/s11136-022-03185-0.

## Introduction

Maintaining health of an increasing number of middle-old and oldest-old people is a major challenge for aging societies [1]. Population norms of health-related quality of life (HrQoL) suggest that HrQoL decreases with age and drops considerably beyond the age of 75 [2, 3]. Numerous interventions targeting this population are, therefore, being developed. In the face of scarce resources, new interventions should be economically evaluated before being implemented in the healthcare system, as such information can assist in the efficient allocation of resources.

To make effects comparable across interventions, economic evaluations often measure effectiveness in terms of quality-adjusted life years (QALY), where the ‘Q’ is measured using generic HrQoL instruments. The most frequently used instrument, in general but also for evaluation of interventions targeting the older population, is the EQ-5D [4–6], which is the officially required standard measurement in some countries (e.g., UK [7]). It consists of five questions covering the dimensions mobility, self-care, usual activities, pain/discomfort, and anxiety/depression. Depending on the version of the EQ-5D, each dimension has three (EQ-5D-3L) or five (EQ-5D-5L) severity levels (“no problems” to “extreme problems”). The combined answers can be transformed to an index with 0 representing death and 1 representing the best possible HrQoL. It is important that the EQ-5D is psychometrically sound in the population it is used, meaning that it measures what it intended to measure (validity) in an accurate and reproducible way (reliability) and is able to detect important changes over time (responsiveness). In the absence of sufficient measurement properties, the results of economic evaluations fail in measuring the true effect of interventions and, thus, are not suitable as basis for decision making regarding their implementation.

Previous reviews examined the psychometric performance of the EQ-5D in different population groups. It was found appropriate for depression and personality disorders [8, 9], urinary incontinence [10], some skin diseases [11], and in people aged 60 or older [12]. However, its psychometric performance was lacking in populations with anxiety, schizophrenia, bipolar disorders, or multiple sclerosis [8, 9, 13]. Moreover, it was found insufficiently sensitive to change in a range of disorders [14]. Regarding its use in dementia, the validity was found problematic as there are significant disagreements between patient and proxy ratings and aspects being important for people with dementia are not adequately reflected [15, 16]. Similarly, other authors conclude that the EQ-5D may not be appropriate in other conditions prevalent in the older population, such as hearing impairments, visual disorders, and some cancers [17, 18]. A common problem seems to be that the EQ-5D has limited ability to differentiate between healthier individuals [19]. Although this ceiling effect could be reduced for the EQ-5D-5L, it still exists [20]. Moreover, the EQ-5D has been criticized for its narrow focus of health, which may fall short on or excludes important aspects of health (e.g., social aspects) [21]. As people’s needs and desires change with age, it can be assumed that, especially in old age or at the end of life, such aspects become more important [22–24].

These findings raise questions regarding the measurement properties of the EQ-5D in middle-old and oldest-old people. To our knowledge, there has been no systematic summary of the measurement properties of the EQ-5D in this population. In a review that is more than a decade old, Haywood et al. [12] evaluated the measurement and practical properties of generic health instruments in older people and found evidence for the validity of the EQ-5D. In terms of responsiveness, the EQ-5D appeared to perform well in people with substantial changes in health; however, responsiveness in terms of correlation of change scores between the EQ-5D and other (clinical) measures was rarely addressed until then. In addition to being outdated and hence including only studies using the EQ-5D-3L, this review did not specifically focus on middle-old and oldest-old people. More recent reviews concluded that the EQ-5D has good feasibility properties in an older population [25], but due to its sole focus on health status, may not be appropriate for measuring outcomes in economic evaluation within aged care, especially in interventions that have effects beyond health status [6, 26, 27]. However, the authors focused exclusively on dependent older people and/or did not systematically summarize the measurement properties of the EQ-5D. Therefore, the aim of the current study was to extend the existing literature by synthesizing and critically appraising studies assessing the measurement properties—reliability, validity, or responsiveness—of the EQ-5D in a population of middle-old and oldest-old people (mean age ≥ 75 years).

## Materials and methods

This review was conducted in adherence with the Consensus-Based Standards for the Selection of Health Measurement Instrument (COSMIN) Methodology for Systematic Reviews of Measurement Properties of PROMs [28]. It has been registered with PROSPERO (Registration Number: CRD42020196070), and a study protocol has been published [29]. The manuscript was prepared based on the Preferred Reporting Items for Systematic Reviews and Meta-Analysis (PRISMA) checklist (electronic supplementary material [ESM] 1) [30].

### Eligibility criteria

Cross-sectional or observational studies providing empirical evidence of reliability, validity, and/or responsiveness of the EQ-5D in a sample with a mean age of ≥ 75 years were included. Studies had to be published in peer-reviewed journals in German or English languages. Systematic reviews, studies applying a qualitative design, or not being original research articles (e.g., conference abstracts or comments) were excluded. Furthermore, studies relying on proxy assessments only or those with the single objective of investigating agreement between different modes of administration of the EQ-5D were excluded. The question of inter-rater agreement between the patient and a proxy often concerns people with dementia and has been addressed in previous reviews [15, 16]. No restrictions relating to interventions, health conditions, publication date, or the version of the EQ-5D (3-level or 5-level) were made.

### Data sources and search strategy

PubMed, Web of Science, Cochrane Library, Embase, and EconLit were searched electronically on March 10, 2021 using predefined search terms, including *quality* *of* *life*, *health-related* *quality* *of* *life*, *EQ-5D*, *EuroQoL*, *aged*, elder*, old*, geriatric*, and *ag*(*e*)*ing* and an adapted search filter for finding studies on measurement properties [31]. Search terms covering non-relevant measurement properties were removed from the search filter (e.g., inter-rater reliability or cross-cultural validity). Where possible, search terms were used as keywords in the title/abstract or Medical Subject Headings (MeSH). An example for the search strategy in PubMed is displayed in Table S1 (ESM 1). Additionally, reference lists of included studies were hand searched.

### Selection of studies and data extraction

Search results from all databases were combined in a shared data repository and managed with Endnote X8. After removing duplicates, two independent reviewers (SG and MN) screened the titles and abstracts and assessed the full texts of the selected abstracts for eligibility. In case of disagreement or uncertainty, a third person (JD) was consulted. Using a standardized data extraction sheet, relevant data from the eligible studies were extracted by one reviewer (SG) and cross-checked by the second reviewer (MN). Data extracted from the individual studies included setting/country, population characteristics, type and method of validity, reliability and responsiveness assessment, and results for each measurement property.

### Assessment of study quality

Methodological quality of included studies was assessed by two reviewers (MN and SG) using the COSMIN Risk of Bias checklist, which was developed specifically for the use in systematic reviews of patient-reported outcome measures [32]. It consists of 10 boxes, each referring to a particular measurement property and containing a different number of sub-questions. Each item is rated on a four-point scale (“very good” to “inadequate”). Any disagreements were resolved through discussion with a third person (JD). Risk of bias rating for each study and measurement property are provided in ESM 2.

### Evaluation of measurement properties

Updated criteria for good measurement properties were applied to rate the individual studies’ results as “sufficient” (+), “insufficient” (−), or “indeterminate” (?) [33]. Reliability was considered “sufficient” if the intraclass correlation coefficient (ICC) was ≥ 0.70. Construct validity and responsiveness were rated “sufficient” if the result was in accordance with predefined hypotheses. The hypotheses were formulated by the review team in advance and where partly (but not necessarily) adopted from the authors of the individual studies. Generic hypotheses applied in this study are presented in Table 1. A detailed overview of specific hypotheses for each individual study is provided in Table S2, ESM 1. The hypotheses regarding the discriminative ability of the EQ-5D between relevant subgroups (e.g., known-groups validity or responsiveness) were accepted if the difference between subgroups was clinically relevant, which was considered more important than whether the difference is statistically significant [34]. For the EQ-5D-3L index, a minimally clinically important difference (MCID) of 0.074 was applied, which was identified as the mean MCID across different patient groups [35]. The studies reporting on known-groups validity or responsiveness of the EQ-5D-5L index were either conducted in the UK or used UK value sets. Therefore, an MCID of 0.063 was applied, which was identified as MCID for England [36].

**Table 1:** Generic/general hypotheses for construct validity and responsiveness (adapted from Prinsen et al., [28])

H1	Correlations with (changes in) instruments measuring similar constructs should be high (≥ 0.5)
H2	Correlations with (changes in) instruments measuring related, but dissimilar constructs should be at least moderate (≥ 0.3)
H3	Correlations with (changes in) instruments measuring weakly related constructs should be at least weak (≥ 0.1)
H4	Correlations with (changes in) instruments measuring unrelated constructs should be negligible (< 0.1)
H5	Meaningful changes between relevant (sub) groups. MCID of the EQ-5D: 0.074 (EQ-5D-3L)[35] or 0.063 (EQ-5D-5L) [36]
H6	For responsiveness (criterion approach), AUC should be ≥ 0.7
H7	HrQoL may decreases with age, but not necessarily, given the circumstances that this review focusses only on middle-old to oldest-old people
H8	Higher education level/social class might be associated with higher HrQoL, but not necessarily, since the differences may no longer be present in this age group (in later life, lifestyle factors such as physical activity become more important [103])
H9	Lower cognitive status is hypothesized to be associated with lower HrQoL in institutionalized people and/or people with severe dementia, whereas this association may not be visible in people with mild to moderate dementia or non-institutionalized people [102]

*AUC* area under the curve, *MCID* minimal clinically important differences

### Summary and grading of the quality of evidence

Criteria for good measurement properties were applied to the summarized results from the individual studies on each measurement property by rating each property as “sufficient” (+), “insufficient” (−), “inconsistent” (±), or “indeterminate” (?) [33, 37]. For construct validity and responsiveness, the measurement property was rated “sufficient” when ≥ 75% of the individual studies’ results were in accordance with predefined hypotheses. The results were qualitatively summarized by providing, e.g., a range of correlation coefficients for convergent validity and the percentage of hypotheses accepted. The evidence synthesis was performed separately for the EQ-5D-3L and EQ-5D-5L. If the results were inconsistent, reasons for inconsistency were explored (e.g., different results for different subgroups). If no reason for inconsistency could be identified, the result was rated “inconsistent” and the quality of evidence was not further explored. Due to heterogeneity of the populations included in the individual studies, quantitative pooling of results was not performed.

The quality of evidence was graded as “high,” “moderate,” “low,” or “very low” using a modified GRADE approach [38]. Starting with the assumption of “high quality,” it was downgraded if there was a risk of bias (up to − 3 levels), (unexplained) inconsistency (up to − 2 levels), imprecision (e.g., small sample size; up to − 2 levels), or indirect results. Indirectness was not applied in this study since studies examining the measurement properties in other populations than the population of interest were excluded. Specific criteria for downgrading are described in the COSMIN manual [34].

## Results

### Search results

**Fig. 1:**
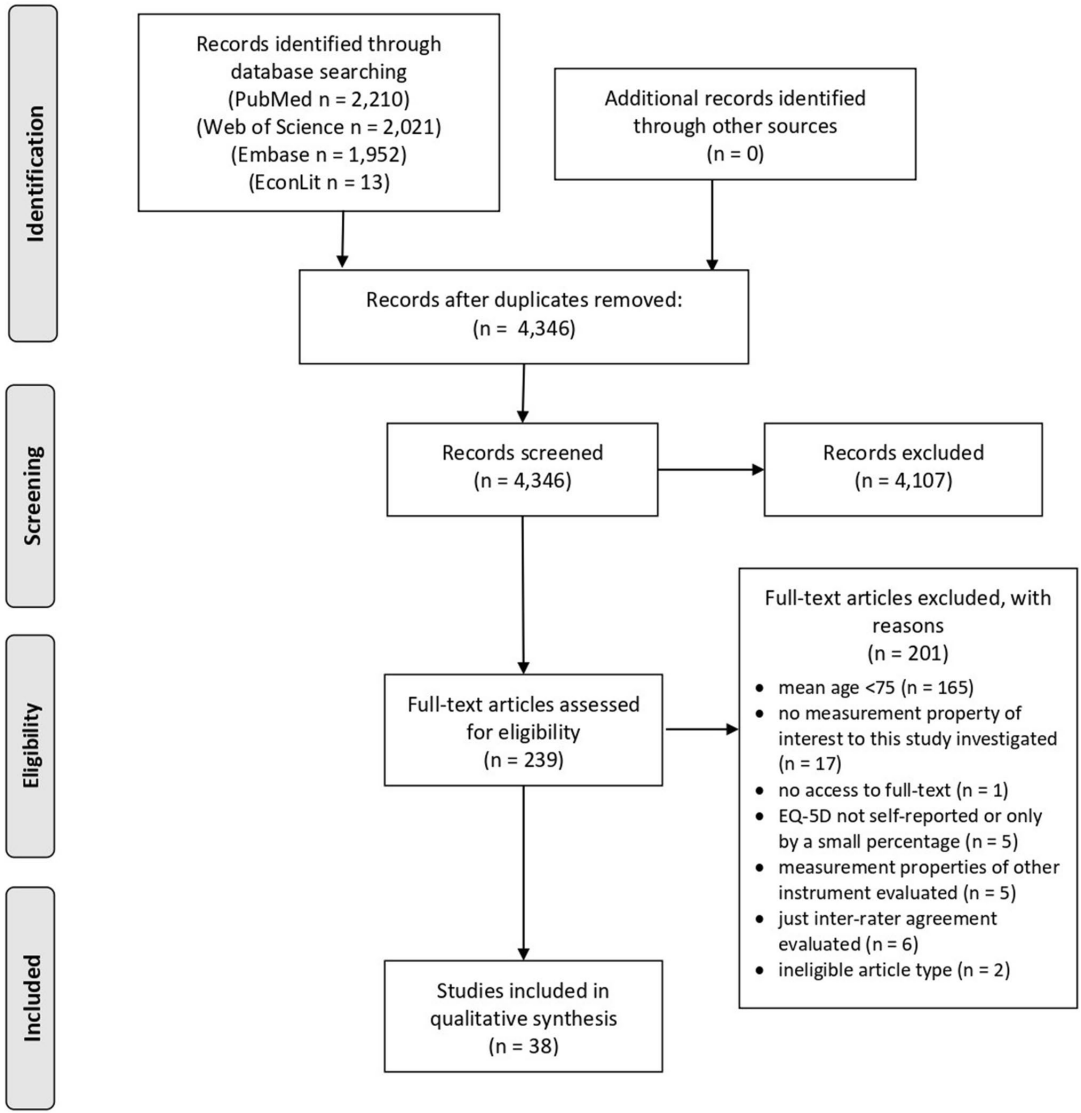
Selection process of included studies

The search strategy resulted in 4346 records (duplicates removed). After screening of title and abstract, 4107 records were excluded, leaving 239 records of which full texts were assessed for eligibility. Finally, 38 records were included for the qualitative synthesis (Fig. 1). No further relevant studies were identified through reference screening. The majority of studies (*n* = 30) evaluated the measurement properties of the EQ-5D-3L [39–68], whereas 9 studies evaluated the EQ-5D-5L [41, 69–76]. One study evaluated both EQ-5D versions [41].

### General characteristics of the articles

Characteristics of the included studies are described in Table 2. Studies covered a variety of (disease) populations, such as people with dementia or cognitive impairment (*n* = 13) [39, 50, 52, 54, 57, 58, 60, 62–64, 69, 72–74], people with different kinds of fractures (*n* = 7) [43, 46, 59, 61, 65, 66, 76], people who were frail or had a history of falling (*n* = 4) [44, 45, 67, 70], or people with venous leg ulcers (*n* = 2) [68, 71]. The studies were conducted in the UK (*n* = 12) [40, 42, 43, 47, 49, 60, 61, 68, 69, 73–75], Sweden (*n* = 3) [59, 65, 66], Spain (*n* = 2) [62, 63], Norway (*n* = 2) [46, 70], Finland (*n* = 1) [48], France (*n* = 1) [39], Germany (*n* = 2) [54, 57], Korea (*n* = 1) [53], the Netherlands (*n* = 2) [55, 67], Australia (*n* = 4) [51, 71, 72, 76], Canada (*n* = 3) [44, 45, 58], the USA (*n* = 2) [52, 56], Mexico (*n* = 1) [64], Sweden/Denmark/Finland/Norway (*n* = 1) [50], or Belgium/Ireland/Netherlands/Switzerland (*n* = 1) [41]. Participants were recruited from different settings, e.g., residential care homes, home-care registries, general practices, falls prevention clinics, or the general population.

**Table 2:** Characteristics of the included studies

Ref	Population			Disease/population characteristics			Instrumental administration	
	N*	Age Mean (SD, range)	% female	Disease/other characteristics of the study population/recruited from	Disease duration	Disease severity	Interview administration mode	Country
**EQ-5D-3L**								
Ankri et al. [39]	142	82.9 (8.3) (60–99)	79.6%	PwD; hospitalized, institutionalized, or outpatients; recruited from geriatric hospital centers	N.R	47.1% moderate, 27.9% severe	Assisted interviews	France
Barton et al. [40]	392	N.R.^1^	N.R	Registered in one general practice	N.A	N.A	N.R	UK
Brazier et al. [42]	377	80.1 (4.5)	100%	Older women, 86.5% long-standing illness or disability; recruited from four general practices into a RCT of clodronate	N.A	N.A	N.R	UK
Coast et al. [43]	214	79^2^ (74–84)^3^	70%	Elderly acute care patients being suitable for rehabilitation in their own home (mainly fractured neck of the femur, elective hip and knee replacements, other fractures, and stroke)	N.A	N.A	N.R	UK
Davis et al. [44]	215	79.3 (6.2)	71.6%	Older adults at risk of mobility impairment and a fall history; visiting the Vancouver falls prevention clinic	N.A	N.A	N.R	Canada
Davis et al. [45]	356	82 (6.5)	63%	Older adults at risk of mobility impairment and a fall history; visiting the Vancouver falls prevention clinic	N.A	N.A	N.R	Canada
Frihagen et al. [46]	222 (complete cases at follow-up: *n* = 79)	82.8 (7.48)	74%	Patients with displaced femoral neck fracture	N.R	*n* = 23 complication group, *n* = 56 non-complication group	Outpatient clinic and home visits	Norway
Hazell et al. [47]	721	N.R.^1^	N.R	Registered in two general practices	N.A	N.A	Postal questionnaire	UK
Heiskanen et al. [48]	36	N.R.^1^	N.R	Patients admitted for CABG operation	N.A	> 60% Canadian cardiovascular society class 3 or 4 (indicating lower functional status)	As part of the preoperative hospital admission process (baseline) and via postal survey (follow-up)	Finland
Holland et al. [49]	145	84.7	57%	People taking ≥ 2 medications; admitted to hospital as an emergency; to be discharged from hospital and returning home/to a warden control accommodation	N.A	Median of 6 daily drugs	Assisted (baseline) interviews	UK
Jönsson et al. [50]	272	75.9	62.3	PwAD, recruited among patients attending regular visits at memory clinics; community dwelling or in residential care	Diagnosed on average 1.6 years prior to inclusion	MMSE > 25 to MMSE < 10	N.R	Sweden, Denmark, Finland, Norway
Kaambwa et al. [51]	87	80 (65–93)	66%	Receiving community aged care services, but cognitively intact	N.A	N.A	Group setting in central venues (research team just there for clarification of questions)	Australia
Karlawish et al. [52]	93	76.8 (2.7) (55–91)	45%	PwAD, not living in care homes, receiving CG assistance; recruited from geriatric medicine practice or memory clinic	N.R	Mild to moderate AD (71% very mild)	Assisted interviews at the participant’s/CG’s home or other convenient location	USA
Kim et al. [53]	2826	N.R.^1^	0%	General male adult population sample, participating in Korean community health survey	N.A	57% moderate to severe lower urinary tract symptoms	Face-to-face interviews	
Kunz [54]	390	80.2 (6.7) (65–100)	68%	PwD, living at home and supported by a family caregiver; recruited via general practitioners	N.R	Mild to moderate	Assessed at GP practices by trained GP and medical secretary	Germany
Lutomski et al. [55]	25,637	78 (6)	58.3%	Community-dwelling older persons aged 65+; recruited from primary care centers, hospitals, or the general population	N.A	73% with hearing issues, joint damage, urinary incontinence, and/or dizziness with falls	N.R	Netherlands
Malkin et al. [56]	77	77^2^ (27–98)	71%	Low-vision patients; presenting for low-vision rehabilitation at private outpatient centers	N.R	55% macular disorders	Telephone interview before 1st visit at low vision rehabilitation site	USA
Michalowsky et al. [57]	560	79.03 (8.5)	59.1%	PwD, living in the community; supported by informal CG	N.R	N.R	Face-to-face interview	Germany
Naglie et al. [58]	57	78.6 (53.8–93.7)	61.7%	PwAD, supported by a family CG; recruited from dementia clinics & geriatric practices	N.R	Mild to moderate	Assisted interview at participant’s home/referring clinic	Canada
Olerud et al. [59]	145	74.7 (9.6) (55–93)	84%	Patients with acute proximal humeral fracture; living non-institutionalized; no severe cognitive dysfunction	N.A	2-part to 4-part fractures	N.R	Sweden
Orgeta et al. [60]	478	75.5 (7.3)	49.6%	PwD living in the community; supported by carers assisting with ADL	N.R	Mild (74.6%) to moderate (25.4%) dementia	Assisted interview	UK
Parsons et al. [61]	225 (sample 1) 249 (sample 2)	83.1 (7.94) 83.6 (7.77)	71% 75%	Patients with hip fracture	N.A	32% and 41% PwD	Face to face at BL; telephone interview at 4 week, 4 month & 12 month FU	UK
Pérez-Ros and Marínez-Arnau [62]	251	84.6 (9.22) (70–104)	76.9%	Nursing home residents with cognitive impairment	N.A	Mean MMSE score: 15.6 (5.23)	Face-to-face interview	Spain
Pérez-Ros et al. [63]	188	79.19 (5.18) (70–95)	64.9%	Community-dwelling older people with cognitive impairment	N.A	MMSE scores 10–24	Face-to-face interview	Spain
Sanchez-Arenas et al. [64]	109	78.5 (7.09)	64.2%	PwD; community dwelling	N.A	N.R	In-home face-to-face interview	Mexico
Tidermark et al. [65]	110	80.0 (70–96)	79%	People with acute displaced femoral neck fractures; no severe cognitive impairment	Fractures ≤ 24 h. old	51% total hip replacement, 49% internal fixation	Structured interview at initial hospital stay and at 4 months	Sweden
Tidermark & Bergström [66]	59	82.9 (5.4) (70–92)	100%	Women with acute femoral neck fractures; no severe cognitive impairment	Fractures ≤ 24 h. old	N.A	Structured interview at initial hospital stay and at 6 months	Sweden
van Leeuwen et al. [67]	190	82.4 (7.7)	71.6%	Frail older adults living at home; recruited from general practices	N.A	N.A	Interview at participants’ homes	Netherlands
Walters et al. [68]	233	75^2^	66.5%	People with venous leg ulcers	present for ≥ 3 months	N.R	Community clinic setting	UK
**EQ-5D-5L**								
Aguirre et al. [69]	272	82.6 (8.1) (52–100)	61%	PwD; recruited from community (58%) and care homes (42%)	N.R	Mild to moderate	N.R	UK
Bjerk et al. [70]	155	82.7 (6.7)	79.3%	Older home-care recipients; experienced ≥ 1 fall in previous 12 months, not cognitively impaired; recruited from lists of people receiving professional home care	N.A	N.A	Interviewed in participants’ homes by trained research assistants	Norway
Cheng et al. [71]	80	75 (13.88) (30–95)	59%	People with venous leg ulcers; recruited from 2 communities, 1 specialist & 1 hospital outpatient wound clinic	0–369 months	Venous insufficiency (41%), reduced mobility (81%)	N.R	Australia
Easton et al. [72]	143	85.7 (8.8) (49–99)	72%	Residential care setting	N.A	45% mild or moderate cognitive impairment, 25% dementia	N.R	Australia
Griffiths et al. [73]	726 (377 completed self-report measures)	85.6 (7.64) (57–102)	73.8%	PwD, living in care homes	N.R.	N.R	N.R	UK
Martin et al. [74]	1004	85.5 (58–103)	73.2%	PwD, living in residential care	N.A	N.A	Recruited from care homes	UK
Nikolova et al. [75]	1038	N.R (75% 75–84, 25% 85+)	52.7%	Community-dwelling older people; recruited from general practices	N.A	20.2% fit, 51.4% pre-frail, 28.4% frail	Face-to-face interview	UK
Ratcliffe et al. [76]	240 (EQ-5D self-completed: *n* = 82)	88.6 (5.6)	74.2%	Patients with hip fracture, living in residential aged care; recruited from acute orthopedic wards	N.A	93% moderate/severe dementia	N.R	Australia
**EQ-5D-3L & EQ-5D-5L**								
Bhadhuri et al. [41]	224	77.5 (5.35)	43.8%	People with multimorbidity and polypharmacy participating in a structured medication review RCT	N.A	N.A	Telephone interview	Belgium, Ireland, Netherlands, Switzerland

*N.R* not reported, *N.A.* not applicable, *ADL* activities of daily living, *BL* baseline, *CG* caregiver, *FU* follow-up, *GP* general practice/practitioner, *MMSE* mini mental state examination, *Pw(A)D* people with (Alzheimer's) dementia, *RCT *randomized controlled trial

*Sample size may differ for specific analyses, ^1^only results for age group ≥ 75 years (or ≥ 80 years [53]) were extracted and included in this review, ^2^median, ^3^inter-quartile range

### Evidence synthesis (Measurement properties)

The summarized results are presented in Table 3 (EQ-5D-3L) and Table 4 (EQ-5D-5L).

**Table 3:** Summary of findings—EQ-5D-3L

Measurement property	Summary	Overall rating	Quality of evidence
**Reliability**	Sub-dimensions [39]: Kappa: 0.34–0.59 (*n* = *45)*	–	**very low**
	Index: *ICC* = 0.58–0.79 [39, 52, 58, 67], *r* = 0.67 [42] (*n* = *439)*	±	**N/A**
**Construct validity**			
**Convergent validity**		**+ (91%)**	**high**
HrQoL instruments (*Hypothesis*: *r* ≥ 0.5)	SF-6D [40], SF-36 [65], HUI3 [58], 15D [48]:** 0.44** [48]–0.74; SF-12 MCS [67]: 0.36^a^ ; SF-12 PCS [67]: 0.60 (*n* = *633* *or* *higher* (*n.c.r*. [58]))	+ (83%)	high
QoL instruments (*Hypothesis*: *r* ≥ 0.3)	ICECAP-O [44, 61, 67], OPQOL-Brief [51], ASCOT [51, 67], AQoL [49], QWB scale [58], QoL-AD [50, 57]: 0.34–0.73 (*n≈1,588* (*n.c.r*. [50, 57, 58, 61]))	+ (100%)	high
General health/QoL (single-scale) (*Hypothesis*: *r* ≥ 0.3)	Health GRS [67], EQ-VAS [39, 50, 58, 63], QoL GRS [67], Cantril’s Self-Anchoring ladder [55], SF-36 general health [58], other [57] (3-pt ordinal scale): 0.34–0.52 (*n≈27,978* (*n.c.r*. [50]))	+ (100%)	high
ADL (*Hypothesis*: *r* ≥ 0.3)	Barthel [54, 62], Katz [58, 64, 67]: **0.25** [58]–0.71; Bristol Activities of Daily Living Scale [60]: *β* = − 0.257 (*n* = *1356* *or* *higher* (*n.c.r*. [58]))	+ (86%)	moderate
IADL (*Hypothesis*: *r* ≥ 0.1)	Lawton & Brody [44, 58, 62], other [64], NOSGER [54]:** 0.03** [44], 0.22–0.62 (*n* = *904* *or* *higher* (*n.c.r* [58]))	+ (80%)	moderate
Comorbidities (*Hypothesis*: *r* ≥ 0.1)	Charlson [64], other [58] (0, 1, ≥ 2): 0.30–0.36 (*n* = *102* *or* *higher* (*n.c.r*. [58]))	+ (100%)	high
Cognitive status/dementia severity (*Hypothesis*: *r* < 0.3)	MMSE [44, 50, 54, 58, 64]: 0.07–0.20 (*n≈1,000* (*n.c.r*. [50, 58]))	+ (100%)	moderate
Depression/anxiety (*Hypothesis*: *r* ≥ 0.1)	GDS [57, 58, 62]: **0.042** [62], 0.21–0.55; CSDD [60]: ***β*** **=** **−** **0.065** (*p* > 0.05); RAID [60]: *β* = − 0.168 (*n≈1,280* (*n.c.r*. [57, 58]))	± (60%)	N/A
Other instruments	*n≈770* (*n.c.r*. [61])	+ (100%)	high
(*Hypothesis*: *r* ≥ 0.3)	OHS [61]: 0.70–0.77		
(*Hypothesis*: *r* ≥ 0.1)	Pearlin Mastery Scale [67], Tinetti [62], VAS Pain [62]: 0.17–0.33		
(*Hypothesis*: *r* < 0.3)	CCCQ [67], PPA [44], SPPB [44]: 0.01–0.06		
**Known-groups validity**	*n≈31,176* (*n.c.r*. [49, 53, 54, 57])	**± (67%)**	**N/A**
*Supported* *for* *groups* *of…*	Age^b^ [42, 43, 49, 55, 68], sex [49, 51], social class^b^ [49], education level^b^ [51, 55], general health [51, 52, 57], mental & physical functioning (SF-12) [52], QoL-AD Score/Whole/Memory [52], IADL impairment (Lawton & Brody) [52, 57], disability severity [42], walking ability [68], number of medications [49], lower urinary tract symptom severity [53], obstructive airways disease (y/n) [47], depression (GDS) [52, 57], hospital stay (y/n) [42], multimorbidity [55], longstanding illness (y/n) [42], cognition (MMSE)^b^ [52], confusion (mental test score)^b^ [49], memory problems (GDS Memory)^b^ [52],		
*Rejected* *for* *groups* *of…*	Age^b^ [51], sex [55], living situation (alone vs. not alone/other arrangement) [49, 51, 55], informal care support (y/n) [51], marital status [55], GP visit (y/n) [42], outpatient attendance (y/n) [42], accident/emergency department attendance (y/n) [42], ADL impairment (higher vs. lower, Lawton-Brody) [52], only dementia vs. dementia + additional comorbidity [54], leg ulcer size and duration [68], functional impairment due to dementia [57], QoL-AD Life [52]		
**Responsiveness**			
**Construct approach**		**− (22%)**	**high**
HrQoL instruments (*Hypothesis*: *r* ≥ 0.5)	SF-36 [65], NHP [66], SF-12 PCS [67], 15D^c^ [48]: **0.23–0.39;** SF-12 MCS^a^ [67]: **0.02** (*n* = *430)*	− (0%)	high
QoL instruments (*Hypothesis*: *r* ≥ 0.3)	ICECAP-O [67], ASCOT [67]: **0.01–0.09;** AQoL [49]: 0.48 (*n≈219* (*n.c.r*. [49]))	± (33%)	high
General health/QoL (single-scale) (*Hypothesis*: *r* ≥ 0.3)	Health GRS [67], QoL GRS [67]: 0.12–0.14 (*n* = *149)*	− (0%)	high
ADL (*Hypothesis*: *r* ≥ 0.3)	Barthel [54], Katz [67]: **0.04–0.19** (*n* = *484)*	− (0%)	moderate
IADL (*Hypothesis*: *r* ≥ 0.1)	NOSGER [54]: **0.01** (*n* = *336)*	− (0%)	high
Cognitive status/dementia severity (*Hypothesis*: *r* < 0.3)	MMSE [54]: 0.00 (*n* = *369)*	+ (100%)	low
Other instruments	*n* = *371*	± (50%)	N/A
(*Hypothesis*: *r* ≥ 0.3)	DASH [59]: 0.47		
(*Hypothesis*: *r* ≥ 0.1)	Pearlin Mastery Scale [67], Activity inventory [56]: **0.02–0.06**		
(*Hypothesis*: *r* < 0.3)	CCCQ [67]: 0.09		
**Comparison between subgroups**	*n≈1,711* (*n.c.r*. [54])	**+ (79%)**	**moderate**
*Supported* *for* *groups* *of…*	Improvement/worsening on the Barthel index [41], knee replacement vs. femur fracture [43], femur fracture vs. stroke [43], fallers vs. non-fallers [45], complication vs. non-complication after femoral neck fracture [46], deterioration in health status (CGI-I) [54], less good vs. good outcome after femoral neck fracture (pain and/or needing walking aids) [65], perceived health change and healing status in people with venous leg ulcers [68], complications/non-complications after femoral neck fracture [46], improvement/deterioration status (DASH) after proximal humeral fracture [59], death/non-death after hip fracture [61], displaced/undisplaced femoral neck fractures [66]		
*Rejected* *for* *groups* *of…*	Improvement/worsening on the EQ-VAS [41], hip replacement vs. femur fracture [43]; healed vs. non-healed leg ulcers at 3 months follow-up [68], revision after hip fracture [61]		
**Before and after intervention**			
*Supported* *for…*	Deterioration/improvement of HrQoL over time after hip or proximal humeral fracture [59, 61] (*n* = *340)*	+ (100%)	high
*Rejected* *for* *…*	Low-vision rehabilitation [56] (*n* = *77)*	– (0%)	moderate

Unless otherwise indicated, reported numbers refer to absolute correlation coefficients, correlation coefficients printed in **bold** indicate results for which the hypotheses were rejected

*y/n* yes/no, *n.s.* not significant, *N/A* not applicable, *N/R* not reported, *r* correlation coefficient, *β* regression coefficient, *n* sample size, *n.c.r.* not clearly reported, *ADL* activities of daily living, *ASCOT* adult social care outcomes toolkit, *AQoL* assessment of quality of life, *CCCQ* client-centered care questionnaire, *CGI-I* clinical global impression of improvement, *CSDD* Cornell Scale for depression in dementia, *DASH* disabilities of arm, shoulder, and hand, *EQ-VAS* Visual Analogue Scale, *GDS* Geriatric Depression Scale, *GRS* Global Rating Scale, *HrQoL* health-related quality of life, *HUI3* Health Utilities Index, *IADL* instrumental activities of daily living, *ICC* intraclass correlation coefficient, *ICECAP-O* ICEpop CAPability measure for older people, *MCS* mental health component summary, *MMSE* mini-mental state examination, *NHP* Nottingham Health Profile, *NOSGER* Nurses’ Observation Scale for Geriatric Patients, *OHS* oxford hip score, *OPQOL-Brief* older people’s quality-of-life brief questionnaire, *PCS* physical health component summary, *PPA* physiological profile assessment, *QoL* quality of life, *QoL-AD* quality of life in Alzheimer’s diseases, *QoL* *GRS* Quality-of-Life Global Rating Scale, *QWB* quality of well-being, *RAID* Rating of Anxiety in Dementia Scale, *SF-36* 36-item short-form health survey, *SF-12* 12-item short-form health survey, *SF-6D* six-dimensional short form, *SPPB* short physical performance battery, *VAS* *Pain* visual analogue scale for pain

^a^deviating hypothesis: *r* ≥ 0.1

^b^no relevant difference between groups hypothesized

^c^no calculation of correlation, instead comparison of EQ-5D & 15D in terms of proportions of changes stratified according to the minimally important difference values

**Table 4:** Summary of findings—EQ-5D-5L

Measurement property	Summary or pooled results	Overall rating	Quality of evidence
**Reliability**	N/R		
**Construct validity**			
**Convergent validity**		**+ (84%)**	**High**
HrQoL instruments (*Hypothesis*: *r* ≥ 0.5)	SF-6D: 0.71 [70], *ICC* = 0.61 [75] (*n≈1193* (*n.c.r*. [75]))	+ (100%)	High
QoL instruments (*Hypothesis*: *r* ≥ 0.3)	DEMQOL [69], DEMQOL-U [72], QOL-AD [69, 73]: 0.30–0.48 QOL-AD-NH [74]: **0.28;** SPVU-5D [71]: *ICC* = 0.55 (*n≈1417* (*n.c.r*. [71]))	+ (83%)	High
General health/QoL (single scale) (*Hypothesis*: *r* ≥ 0.3)	EQ-VAS [71]: 0.39 (*n≈75* (*n.c.r.*))	+ (100%)	Moderate
ADL (*Hypothesis*: *r* ≥ 0.3)	MBI [72, 76]: 0.46–0.49 (*n* = *225)*	+ (100%)	High
Cognitive status (*Hypothesis*: *r* < 0.3)	Pas-Cog* [72]: **0.24**; MMSE [76]: 0.22; CDR [74]: 0.025 (*n* = *1116)*	± (67%)	N/A
Other instruments	*n* = *1113*	+ (80%)	High
(*Hypothesis*: *r* ≥ 0.1)	CSDD [76], PainAd [76]: 0.33–0.45; FAST [74]: **0.049**		
(*Hypothesis*: *r* < 0.3)	CMAI [74], NPI-Q [72]: 0.1		
**Known-groups validity**	*n≈306* (*n.c.r*. [71])	**+ (78%)**	**High**
*Supported* *for* *Groups* *of…*	Age^a^ [71], general health (EQ-VAS) [71], leg ulcer healing status [71], physical functioning/ADL (MBI) [72, 76], pain (PainAd) [76], depression (CSSD) [76]		
*Rejected* *for* *groups* *of…*	cognitive impairment (PAS-Cog)* [72], ulcer duration [71]		
**Responsiveness**			
**Construct** **approach**		**+ (75%)**	**High**
QoL instruments (*Hypothesis*: *r* ≥ 0.3)	QOL-AD-NH [74]: ***β*≈0.007** (*p* < 0.05) (*n≈261*(*n.c.r.*))	− (0%)	Moderate
Cognitive status (*Hypothesis*: *r* < 0.3)	CDR [74]: *β* = n.s (*n≈261*(*n.c.r.*))	+ (100%)	High
Other instruments	*n≈396* (*n.c.r.* [74])	+ (83%)	High
(*Hypothesis*: *r* ≥ 0.3)	BBS [70]: Elasticity = 0.54		
(*Hypothesis*: *r* ≥ 0.1)	30 s STS [70], 4 m walk test [70], FES-I [70]: Elasticity = 0.09–0.24; FAST [74]: ***β*** **=** **n.s**		
(*Hypothesis*: *r* < 0.3)	CMAI [74]: *β* = n.s		
** Comparison between subgroups**	*n* = *269*	**+ (75%)**	**High**
*Supported* *for* *groups* *of…*	Improvement/worsening on the Barthel index [41], healing status and duration of venous leg ulcers [71]		
*Rejected* *for* *groups* *of…*	Improvement/worsening on the EQ-VAS [41]		
**Before and after intervention**	N/R		

+ sufficient, − insufficient, ± inconsistent, *y/n* yes/no, *n.s.* not significant, *r* correlation coefficient, *β* regression coefficient, *n* sample size, *n.c.r.* not clearly reported, *N/R* not reported, *BBS* Berg Balance Scale, *CDR* clinical dementia rating, *CMAI* Cohen-Mansfield Agitation Inventory, *CSDD* Cornell Scale for Depression in Dementia, *DEMQOL* dementia quality of life, *EQ-VAS* Visual Analog Scale, *FAST* functional assessment staging, *FES-I* Falls Efficacy Scale International, *ICC* intraclass correlation coefficient, *MBI* Modified Barthel Index, *MMSE* mini-mental state examination, *PainAd* Pain Assessment in Advanced Dementia Scale, *PAS-Cog* Psychogeriatric Assessment Scales-Cognitive Impairment Scale, *QoL* quality of life, *QoL-AD* quality of life in Alzheimer’s disease, *QOL-AD-NH* quality of life in Alzheimer’s disease nursing home version, *SF-6D* six-dimensional short-form health survey, *30* *s* *STS* 30-second sit-to-stand test, *SPVU-5D* five-dimensional sheffield-preference-based venous ulcer questionnaire

*result in the opposite of the hypothesized direction (H9)

^**a**^no relevant difference between groups hypothesized

#### Reliability

In total, five studies assessed the reliability of the EQ-5D-3L index, with three reporting sufficient [39, 58, 67] and two reporting insufficient reliability [42, 52]. In one of the two studies of insufficient reliability [42], the time interval between measurements (6 months) was inappropriate (doubtful methodological quality). However, for the other study with insufficient reliability [52], no possible explanation could be found (similar population and/or time interval like in other studies reporting sufficient reliability [39, 58]). Thus, the overall rating of reliability of the EQ-5D-3L was inconsistent. Very low-quality evidence regarding the reliability of the individual dimensions of the EQ-5D-3L was available from one study [39], which found insufficient reliability based on Kappa coefficients between 0.34 and 0.59.

No study regarding the reliability of the EQ-5D-5L could be identified.

#### Convergent validity

Overall, convergent validity for both EQ-5D versions was supported by multiple studies, with the majority of hypotheses being supported at moderate to high quality of evidence.

As hypothesized, strong correlations between the EQ-5D-3L index and other instruments of HrQoL (SF-12, SF-6D, SF-36, HUI3) were found [40, 58, 65, 67]. At least moderate correlations were found with instruments of QoL (ICECAP-O, OPQOL-Brief, ASCOT, AQOL, QWB, QoL-AD) [44, 49–51, 57, 58, 61, 67], activities of daily living (ADL) (Barthel, Katz, BADL) [54, 58, 62, 64, 67], or single-scale instruments of general health or QoL [39, 50, 55, 57, 58, 63, 67]. Moreover, at least weak correlations with instruments of instrumental activities of daily living (IADL) (e.g., Lawton-Brody, NOSGER) [44, 54, 58, 62, 64] and comorbidities [58, 64] were found in the majority of studies. Results were inconsistent regarding the convergent validity of the EQ-5D-3L index with measures of depression/anxiety, which were hypothesized to be at least weakly correlated [57, 58, 60, 62].

Similarly, the EQ-5D-5L index was strongly correlated with the SF-6D as measure of HrQoL [70, 75]. At least moderate associations were found with QoL instruments (DEMQOL, DEMQOL-U, QOL-AD, SPVU-5D) [69, 71–73] (with the exception of the QoL-AD-NH [74]), as well as with a single-scale instrument for general health (EQ-VAS) [71] or a measure of ADL (MBI) [72, 76]. Results were inconsistent for associations with measures of cognitive status (Hypothesis 9, Table 1) [72, 74, 76], where one study found a positive correlation, although an association in the opposite direction was hypothesized [72].

Several studies [39, 41, 43, 44, 50, 51, 55, 56, 62–64, 68, 70–72, 75] also assessed convergent validity by correlating the EQ-5D index with the individual dimensions of the comparator instrument, the EQ-5D dimensions with a comparator instrument’s summary score, or the EQ-5D dimensions with the comparator’s dimensions (Tables S3 & S4, ESM 1). For both EQ-5D versions, the majority of results were in accordance with the hypotheses, thus, supporting the overall rating of convergent validity as sufficient.

#### Known-groups validity

Twelve studies assessed known-groups validity of the EQ-5D-3L index in a variety of populations [39, 42, 43, 47, 49, 51–55, 57, 68]. Overall, known-groups validity was inconsistent as < 75% of the results (67%) were in accordance with the hypotheses.

For the EQ-5D-5L index, known-groups validity was assessed in three studies [71, 72, 76]. The overall result was rated sufficient (78% of the hypotheses supported) and the quality of evidence was rated high.

Detailed information about the groups that the EQ-5D-3L and EQ-5D-5L were able to discriminate between can be found in Tables 3 & 4.

#### Responsiveness

Eight studies assessed responsiveness of the EQ-5D-3L index by examining the associations of change scores with other instruments [48, 49, 54, 56, 59, 65–67]. With one exception (AQoL) [49], the correlations with changes in instruments of HrQoL (SF-36, SF-12, NHP, 15D) [48, 65–67], QoL (ICECAP-O, ASCOT) [67], single-scale instruments of general health or QoL [67], ADL (Barthel, Katz) [54, 67], and IADL (NOSGER) [54] were weaker than hypothesized. Thus, responsiveness based on the comparison with other instruments was rated insufficient, and the summarized quality of evidence was rated high.

Ten studies assessed responsiveness of the EQ-5D-3L index based on comparisons between subgroups [41, 43, 45, 46, 54, 59, 61, 65, 66, 68]. These studies were primarily conducted on specific patient populations and assessed, e.g., the ability of the EQ-5D to differentiate between different outcomes after fractures or venous leg ulcers. Overall, moderate-quality evidence for sufficient responsiveness of the EQ-5D-3L based on comparisons between subgroups was found, as 79% of the hypotheses were supported.

Three studies [56, 59, 61] examined responsiveness by testing hypotheses regarding change in the EQ-5D-3L index in response to an intervention. Two hypotheses regarding the improvement or deterioration of HrQoL after fracture were supported, whereas, opposed to the hypothesis, low vision rehabilitation did not change HrQoL.

For the EQ-5D-5L index, two studies [70, 74] assessed responsiveness based on comparisons with other instruments. 75% of the results were in accordance with the hypotheses and, thus, were rated as sufficient at high quality of evidence. The correlations of change scores were as high (or low) as hypothesized between the EQ-5D-5L and measures of cognitive status or agitation (CDR, CMAI) [74], measures of physical function (BBS, 30 s STS, 4 m walk test) [70] but were lower than hypothesized between the EQ-5D-5L and a QoL instrument (QOL-AD-NH) [74] or a measure of functional symptoms in dementia (FAST) [74].

Two studies examined responsiveness of the EQ-5D-5L index in terms of subgroup comparisons [41, 71]. 75% of the hypotheses were supported and, thus, the overall result was sufficient. The quality of evidence was rated high.

#### Results not included in the qualitative synthesis

Some results were not included in the qualitative synthesis as no specific results (e.g., correlation coefficients) were reported. Regarding convergent validity, Michalowsky et al. [57] found a poor association (not further specified) between the EQ-5D-3L index and IADL. Other authors examined the association between the EQ-5D dimensions with ADL and found significant associations between several dimensions but did not provide information about the strength of the association [39, 43]. Moreover, the authors assessed known-groups validity and found, e.g., that women were more anxious than men [39] and that people with disability had lower HrQoL than people with no disability [43]. However, it could not be evaluated whether the differences were clinically important because the mean EQ-5D of each group was not reported.

## Discussion

The current study synthesized reliability, validity, and responsiveness of the EQ-5D in a population of middle-old and oldest-old people. Regarding reliability, results were inconsistent for the EQ-5D-3L, and for the EQ-5D-5L, studies were entirely lacking. This may pose a problem in contexts where the EQ-5D is used at different time points to quantify a ‘true’ difference or change in HrQoL, such as in economic evaluations. Previous reviews report mixed results on the reliability of the EQ-5D in people with dementia (moderate to strong) [16] and sufficient reliability in people with diabetes or stroke [77, 78]. Another review further suggests sufficient reliability of the EQ-5D-5L in various patient groups (e.g., osteoarthritis, diabetes and cancer patients, cardiovascular and liver diseases) and general population samples [79]. However, so far, the evidence on reliability for both the EQ-5D-3L and EQ-5D-5L is relatively limited and entirely lacking for certain patient groups.

For both EQ-5D versions, high-quality evidence of sufficient convergent validity was found. It should be noted that high correlations with other generic instruments (e.g., SF-36/-12, SF-6D, HUI3) do not necessarily support the use of the EQ-5D in middle-old to oldest-old people, as it does not preclude that both instruments do not capture aspects that are important to the population of interest. In some cases, convergent validity was assessed by correlations with instruments which were collected only in a single, specific study (e.g., OHS, Pearlin Mastery Scale). These results summarized as “other instruments” despite measuring different constructs in Table 3 and 4, may not be generally relevant for the population aged 75+ but were mostly in accordance with the hypotheses.

Known-groups validity of the EQ-5D-3L was inconsistent. One potential explanation could be a ceiling effect of the EQ-5D-3L, which may have compromised its ability to discriminate between known groups. Moreover, it can be questioned whether the groups for evaluating known-groups validity are relevant (e.g., marital status, living alone vs. not alone). Similarly, it could be questioned whether it is reasonable to examine, e.g., convergent validity of the EQ-5D with instruments measuring constructs which are hardly related to HrQoL (e.g., CCCQ, PPA, SPPB). The evaluation of measurement properties should be theory driven and not exploratory by using all available variables from studies that were initially designed for a different purpose. More precise preliminary hypotheses of associations between measures in studies analyzing an instrument’s measurement properties would, therefore, be desirable. In addition, rather “soft” hypotheses regarding the strength of the association between two instruments were defined in this review, e.g., by not setting an upper limit for correlations between instruments measuring related but dissimilar constructs (*r* ≥ 0.3) or weakly related constructs (*r* ≥ 0.1). This was done to avoid “penalizing” relatively strong correlations between instruments that were assumed to be not necessarily but potentially highly correlated (e.g., EQ-5D and ADL instruments). Since, according to the COSMIN methodology, the synthesized evaluation of a measurement property is based on a majority principle (≥ 75% of the hypotheses supported), these aspects could have influenced the (synthesized) results. For the EQ-5D-5L, high-quality evidence of sufficient known-groups validity was found. There, the selection of groups that the EQ-5D was expected to differentiate between seemed to be less arbitrary, but overall, the results were based on only three studies. The COSMIN methodology recommends judging an instrument’s ability to discriminate between relevant groups based on clinically important rather than statistically significant differences [34]. While being aware that there is no single MCID for EQ-5D index values since it varies by population characteristics [80], in the absence of specific MCIDs for each country-specific tariff and disease group of the individual studies included in this review, MCIDs commonly used in previous literature were nevertheless used but could have influenced the results regarding known-groups validity.

Responsiveness was insufficient (high-quality evidence) for the EQ-5D-3L when correlated with instruments being hypothesized to be related (e.g., other (Hr)QoL instruments). However, it seemed to be responsive to outcomes after fracture or healing status of leg ulcers [43, 46, 59, 61, 65, 66, 68]. These are conditions with substantial changes in health, where the EQ-5D has previously been shown to be more likely to be responsive (in an older population) [12, 18]. Although responsiveness of the EQ-5D-5L (construct approach) was found sufficient according to the majority principle of the COSMIN methodology, the evidence was limited as it was based on only two studies which used very study-specific instruments to evaluate responsiveness (e.g., 30 s STS) [70, 74]. These instruments were hypothesized to be only weakly associated with the EQ-5D and were, therefore, not responsive to changes in HrQoL.

Overall, the results regarding the responsiveness of the EQ-5D suggest that at least the EQ-5D-3L is hardly able to adequately reflect clinical changes over time. In turn, clinically relevant changes may remain undetected; thus, intervention effects may be underestimated based on the EQ-5D. For example, economic evaluations of fall prevention programs showed that clinical effects could not be found on HrQoL [81–83]. This does not seem to be an exclusive problem of the EQ-5D but also of other generic HrQoL instruments, such as the SF-36 or SF-12 [82, 83]. So far, the evidence on responsiveness of the EQ-5D is mainly based on studies using the EQ-5D-3L. The sparse evidence on the responsiveness of the EQ-5D-5L is not limited to the population of middle old to oldest old but is also found in general for other populations [79]. Moreover, the majority of the included studies reported substantial ceiling effects, which may limit the ability to capture small changes at the upper end of HrQoL. Ceiling effects were found to be particularly common among people with dementia [15], who make up a large proportion in the current study. Generally, the EQ-5D-5L was found to reduce this ceiling effect [84, 85]. However, it persists in general population studies but also in some patient populations [79]. Further studies are needed, which evaluate the responsiveness of the EQ-5D-5L to change in, e.g., other (age or disease specific) (Hr)QoL instruments. It would be of particular interest to examine whether the EQ-5D-5L is more responsive than the EQ-5D-3L which was insufficiently responsive in this respect.

The approach to primarily focus on HrQoL in the form of health utility gains in economic evaluations has been criticized for excluding aspects of QoL beyond health [23, 86]. Furthermore, HrQoL instruments such as the EQ-5D or the SF-12/SF-36 are mainly functioning oriented and, thus, do not reflect the breadth of the concept of health as stated in the WHO definition [21], e.g., social aspects of health fall short or are not assessed differentiated enough. This seems to be especially relevant to older people as it was found that not only health but also social domains are important to their overall QoL [23, 87]. Therefore, other instruments were and are currently being developed, which may provide an alternative or complement to measure (Hr)QoL based on a broader or more comprehensive framework of health or well-being in the future. Some age- or disease-specific QoL instruments exist, and the current study showed that although being moderately to strongly associated with the EQ-5D when assessed at a single time point (sufficient convergent validity), changes on these instruments are not reflected on the EQ-5D (insufficient responsiveness). This suggests that the EQ-5D is not able to capture changes in (Hr)QoL that are important to older people. However, the existing age- or disease-specific instruments differ in domains of (Hr)QoL that are captured [6] and, thus, pose a problem for the comparability of intervention effects across diseases and populations. Moreover, the lack of preference-based value sets for some of these instruments (e.g., for the WHOQOL-OLD, an older people-specific QoL instrument [87]) or value sets being only available for the population in the country where the instruments were developed, impedes their use in economic evaluations. Another recently developed instrument is the PROMIS-29, a health profile measure from the Patient-Reported Outcomes Measurement Information System® (PROMIS®) [88–90] that captures health in a broader sense than the EQ-5D. Although value sets are available for the PROMIS-29 [89–92], they are so far only available for the US. Moreover, the ‘Extending the QALY’ research project is currently developing the EQ-HWB, a broad measure of QoL for use in economic evaluations across health and social care (https://scharr.dept.shef.ac.uk/e-qaly/), and thus, could be a potential alternative to the EQ-5D in the future. However, these age-unspecific instruments carry the risk that scoring algorithms used to derive the utility index are based on the preferences of the general adult populations, whose preferences for health may differ from those of older people [6, 24]. Another research group is seeking to address this issue and is currently developing an instrument for quality assessment and economic evaluation that adequately captures the aspects of quality of life that are important to older people, using a person-centered approach [93, 94]. Consequently, as long as there is no single preference-based generic instrument that comprehensively captures relevant aspects of (Hr)QoL in middle-old and oldest-old people or its use is limited in certain situations (e.g., lack of country/population-specific tariffs), age- or disease-specific instruments should be used as complement to the EQ-5D and help interpreting the results of (cost-)effectiveness analyses (e.g., whether the effects of an intervention are likely to be underestimated).

Beyond these alternative instruments, several “bolt-on” dimensions to the EQ-5D have been proposed and a wide variety of methods have been applied to identify or select relevant bolt-on dimensions [95]. Finch, Brazier, Mukuria, and Bjorner [96] identified hearing, sleep, cognition, energy, and relationships as potentially relevant bolt-on dimensions, and some studies have shown that higher severity levels in the bolt-on dimensions impact the health state values or preferences for the health state [97–99]. Recently, Chen and Olsen [100] proposed vitality, sleep, social relationships, and community connectedness as bolt-on dimensions. They argue that adding these four dimensions would provide a solution to assess HrQoL in a single, brief instrument, but still include all key dimensions of the conceptual map of HrQoL by Olsen and Misajon, [21] and, thus, capture health and well-being more broadly than current EQ-5D instruments. However, to use the additional information from the bolt-on dimensions in economic evaluations, the bolt-on dimension scores would need to be incorporated into the utility index, which would require new valuation studies. Moreover, extensive testing on whether the bolt-on dimensions improve psychometric performance of the EQ-5D would be needed, in general, but also particularly in middle-old and oldest-old people.

A large number of the included studies (*n* = 13) assessed the measurement properties of the EQ-5D in people with dementia or cognitive impairment. As part of the validation, the association between (change in) cognitive status and (change in) the EQ-5D was examined [44, 49, 52, 54, 58, 64, 72, 74, 76]. However, the relationship between cognition and (Hr)QoL seems to be complex [101, 102], which made it difficult to formulate (generic) hypotheses regarding the direction and strength of the association in this study.

This review deliberately did not focus on the comparison of self- and proxy-rated EQ-5D scores and did not consider correlations between the self-rated EQ-5D and proxy-rated other (Hr)QoL instruments in the synthesis. (Hr)QoL is a subjective concept; therefore, it is not surprising that different people evaluate it differently, especially when self-perception is impaired by a condition such as dementia, where proxies typically rate the HrQoL of a person with dementia lower than the person him/herself [15, 16]. It is not possible to determine whose rating is more “correct.” However, it is important to be aware of these variations and to select the administration mode depending on the perspective from which the benefits of an intervention are to be measured.

This study applied the updated COSMIN methodology to systematically review the measurement properties of the EQ-5D in a middle-old and oldest-old population. However, several limitations must be acknowledged. First, only studies which directly aimed to examine the measurement properties of the EQ-5D were included, whereas studies providing indirect evidence on measurement properties (e.g., by correlating the EQ-5D with instruments being hypothetically related) were not included. Second, the generalizability of the results may be limited: although this study was deliberately not restricted to specific populations such as disease groups, it is not clear, whether the results apply to the general population of middle-old to oldest-old adults as, e.g., a large share of the included studies included only people with dementia. Moreover, the results do not exclusively apply to the population aged 75+ as a number of persons < 75 years are also included in some of the studies. To date, there have been few studies focusing exclusively on the population aged 75 years and older, representing a gap in research. Such studies could allow a comparison between the measurement properties of the EQ-5D between younger-old (e.g., aged 60+) and middle-old to oldest-old people, which was not directly possible based on the current data. Finally, the evidence stems exclusively from western, industrialized countries and, therefore, may not be transferable to other countries or regions.

## Conclusion

The results of this systematic review are relevant as improving the care and maintaining the health and QoL of an older population is a political goal in many countries. Thereby, the results may be of interest to decision makers, but also to researchers planning, designing, or evaluating interventions for older people.

Based on the findings of this study, both EQ-5D versions seem to have sufficient convergent validity and may, therefore, be used in cross-sectional studies to assess HrQoL. However, caution is advised when using the EQ-5D to assess change in HrQoL, as the EQ-5D-3L was found to be insufficiently responsive to change (except for conditions with substantial changes in health) and results regarding the reliability were inconsistent. As specifically for the EQ-5D-5L little evidence on reliability and responsiveness is available so far, further research might be needed in this regard. If responsiveness cannot be demonstrated, either using additional disease- or age-specific instruments or considering the use of an alternative, more comprehensive instrument of (Hr)QoL might be advisable, especially for economic evaluations. Promising research is currently underway to develop new, more comprehensive instruments that will better capture the aspects of QoL that are important to older people. However, there is still a long way to go to verify their measurement properties, generate population- and country-specific value sets, and thus, be broadly applicable to economic evaluations.

## Supplementary Information

Below is the link to the electronic supplementary material.

Supplementary file2 (XLSX 9162 kb)

Supplementary file1 (PDF 316 kb)

## Data Availability

All data generated or analyzed during this study are included in this published article and its supplementary information files.

## Abbreviations

ADL: Activities of daily living
ASCOT: Adult Social Care Outcomes Toolkit
AQoL: Assessment of Quality of Life
BBS: Berg Balance Scale
BADL: Bristol Activities of Daily Living Scale
CCCQ: Client-centered Care Questionnaire
CDR: Clinical Dementia Rating
CMAI: Cohen-Mansfield Agitation Inventory
COSMIN: COnsensus-based Standards for the selection of health Measurement INstruments
DEMQOL: Dementia Quality of Life instrument
ESM: Electronic supplementary material
EQ-HWB: EQ Health and Wellbeing instrument
EQ-VAS: EQ-Visual Analogue Scale
FAST: Functional Assessment Staging Tool
HrQoL: Health-related quality of life
HUI3: Health Utilities Index
IADL: Instrumental activities of daily living
ICC: Intraclass correlation coefficient
ICECAP-O: ICEpop CAPability measure for Older people
MBI: Modified Barthel Index
MCID: Minimally clinically important difference
MeSH: Medical subject headings
NHP: Nottingham Health Profile
NOSGER: Nurses’ Observation Scale for Geriatric Patients
OHS: Oxford Hip Score
OPQOL-Brief: Older People’s Quality of Life questionnaire, short version
PPA: Physiological Profile Assessment
PRISMA: Preferred Reporting Items for Systematic reviews and Meta-Analysis
PROMIS: Patient-Reported Outcomes Measurement Information System
QALY: Quality-adjusted life years
QoL: Quality of life
QoL-AD: Quality of Life in Alzheimer’s Disease scale
QOL-AD-NH: Quality of Life in Alzheimer’s Disease in Nursing Homes
QWB: Quality of Well-Being scale
SF-36: 36-item Short-Form health survey
SF-12: 12-item Short-Form health survey
SF-6D: Short Form 6 Dimensions
SPPB: Short Physical Performance Battery
SPVU-5D: 5-Dimensional Sheffield Preference-based Venous Ulcer questionnaire
UK: United Kingdom
US: United States
WHOQOL-OLD: World Health Organization Quality of Life - Older Adults
30 s STS: 30-second Sit-To-Stand test
