# Supplementary material for: Measurement properties of the EQ-5D in populations with a mean age of ≥ 75 years: a systematic review
Source: Qual Life Res. 2022 Aug 1;32(2):307–29. doi: 10.1007/s11136-022-03185-0 (PMC9911506; doi:10.1007/s11136-022-03185-0)
Supplement: Supplementary file 2 — Supplementary file1 (PDF 316 kb) [file 11136_2022_3185_MOESM2_ESM.pdf]

Electronic Supplementary Material 1

Manuscript title: Measurement properties of the EQ-5D in populations with a mean age of ≥75 years: a systematic review.

Authors: Sophie Gottschalk, Hans-Helmut König, Mona Nejad, Judith Dams

Corresponding author: Sophie Gottschalk, Department of Health Economics and Health Services Research, University Medical Center Hamburg-Eppendorf, Martinistraße 52, 20246 Hamburg, s.gottschalk@uke.de

Table S1 *Search strategy in PubMed using an adapted version of the patient-reported outcome measurement filter available on the COSMIN website[1]*

1#	(instrumentation[MeSH Subheading] OR “reproducibility of results”[MeSH Terms] OR reproducib*[Title/Abstract] OR “psychometrics”[MeSH] OR psychometr*[Title/Abstract] OR “discriminant analysis”[MeSH] OR reliab*[Title/Abstract] OR valid*[Title/Abstract] OR “internal consistency”[Title/Abstract] OR (cronbach*[Title/Abstract] AND (alpha[Title/Abstract] OR alphas[Title/Abstract])) OR “item correlation”[Title/Abstract] OR “item correlations”[Title/Abstract] OR agreement[Text Word] OR test–retest [Title/Abstract] OR (test[Title/Abstract] AND retest[Title/Abstract]) OR (reliab*[Title/Abstract] AND (test[Title/Abstract] OR retest[Title/Abstract])) OR intra-rater[Title/Abstract] OR intratester[Title/Abstract] OR intra-tester[Title/Abstract] OR OR intraobserver[Title/Abstract] OR intra-observer[Title/Abstract] OR intraindividual[Title/Abstract] OR intra-individual[Title/Abstract] OR intraparticipant[Title/Abstract] OR intra-participant[Title/Abstract] OR kappa[Title/Abstract] OR kappa’s[Title/Abstract] OR kappa’s[Title/Abstract] OR “coefficient of variation”[Title/Abstract] OR repeatable*[Text Word] OR ((replica*[Text Word] OR repeated[Text Word]) AND (measure[Text Word] OR measures[Text Word] OR findings[Text Word] OR result[Text Word] OR results[Text Word] OR test[Text Word] OR tests[Text Word])) OR concordance[Title/Abstract] OR (infraclass[Title/Abstract] AND correlation*[Title/Abstract]) OR discriminative[Title/Abstract] OR “known group”[Title/Abstract] OR “factor analysis”[Title/Abstract] OR “factor analyses”[Title/Abstract] OR “factor structure”[Title/Abstract] OR “factor structures”[Title/Abstract] OR dimensionality[Title/Abstract] OR subscale*[Title/Abstract] OR “item discriminant”[Title/Abstract]OR “interstate correlation”[Title/Abstract] OR “interstate correlations”[Title/Abstract] OR “individual variability”[Title/Abstract] OR “standard error of measurement”[Title/Abstract] OR sensitive*[Title/Abstract] OR responsive*[Title/Abstract] OR “minimal detectable concentration”[Title/Abstract] OR (small*[Title/Abstract] AND (real[Title/Abstract] OR detectable[Title/Abstract]) AND (change[Title/Abstract] OR difference[Title/Abstract])) OR “meaningful change”[Title/Abstract] OR “minimal important change”[Title/Abstract] OR “minimal important difference”[Title/Abstract] OR “minimally important change”[Title/Abstract] OR “minimally important difference”[Title/Abstract] OR “minimal detectable change”[Title/Abstract] OR “minimal detectable difference”[Title/Abstract] OR “minimally detectable change”[Title/Abstract] OR “minimally detectable difference”[Title/Abstract] OR “minimal real change”[Title/Abstract] OR “minimal real difference”[Title/Abstract] OR “minimally real change”[Title/Abstract] OR “minimally real difference”[Title/Abstract] OR “Item response model”[Title/Abstract] OR IRT[Title/Abstract] OR Rash[Title/Abstract] OR “Differential item functioning”[Title/Abstract] OR DIF[Title/Abstract])
#2	(EQ-5D) OR (EQ5D) OR (EuroQoL)
#3	(aged, 80 and over[MeSH Terms]) OR (aged[MeSH Terms]) OR (elderly[MeSH Terms]) OR (aged[Title/Abstract]) OR (elderly*[Title/Abstract]) OR (older*[Title/Abstract]) OR (geriatric*[Title/Abstract])
#4	#1 AND #2 AND #3
#5	(“addresses”[Publication Type] OR “biography”[Publication Type] OR “case reports”[Publication Type] OR “comment”[Publication Type] OR “directory”[Publication Type] OR “editorial”[Publication Type] OR “festschrift”[Publication Type] OR “interview”[Publication Type] OR “lectures”[Publication Type] OR “legal cases”[Publication Type] OR “legislation”[Publication Type] OR “letter”[Publication Type] OR “news”[Publication Type] OR “newspaper article”[Publication Type] OR “patient education handout”[Publication Type] OR “popular works”[Publication Type] OR “congresses” [Publication Type] OR “consensus development conference”[Publication Type] OR “consensus development conference, nigh”[Publication Type] OR “practice guideline”[Publication Type]) NOT (“animals”[MeSH Terms] NOT “humans”[MeSH Terms])
#6	#4 NOT #5

Table S2 *Specific hypotheses for each individual study*

Reference	Hypotheses
Aguirre et al.[2]	**DEMQOL** → moderate to strong<br><br>**QOL-AD** → moderate to strong
Ankri et al.[3]	*Convergent validity:*<br><br>**VAS** → moderate to strong<br><br>**Katz-ADL** & …<br><br>…EQ-5D mobility → moderate to strong<br><br>…EQ-5D self-care → moderate to strong<br><br>…EQ-5D usual activities → moderate to strong<br><br>…EQ-5D pain/discomfort → weak to moderate<br><br>…EQ-5D anxiety/depression → weak to no association<br><br>**CDR** (dementia severity) → H9 (weak)<br><br>**MMSE** (dementia severity) → H9 (weak)<br><br>**Age** → H7<br><br>*Known-groups validity:*<br><br>3) **Sex** & Anxiety/Depression → women are more anxious than men
Barton et al.[4]	**SF-6D** → strong
Bhadhuri et al.[5]	*Convergent validity (EQ-5D-3L & EQ-5D-5L):*<br><br>**Barthel index** & …<br><br>…EQ-5D mobility → moderate to strong<br><br>…EQ-5D self-care & Barthel index → strong<br><br>…EQ-5D usual activities & Barthel index → moderate to strong<br><br>…EQ-5D pain/discomfort & Barthel index → weak to moderate<br><br>…EQ-5D anxiety/depression & Barthel index → weak to moderate<br><br>*Responsiveness – Comparison of subgroups:*<br><br>Moderate to large effect size for the EQ-5D-5L/-3L in the improved Barthel index group.<br><br>Negative effect size, when there is worsening in the Barthel index.<br><br>Moderate to large effect size for the EQ-5D-5L/-3L in the improved EQ-VAS group.<br><br>Negative effect size, when there is worsening on the EQ-VAS.
Bjerk et al.[6]	*Convergent validity:*<br><br>**SF-6D** → strong<br><br>**SF physical functioning** & …<br><br>…EQ-5D mobility → moderate to strong<br><br>…EQ-5D self-care → moderate to strong

	…EQ-5D usual activities → moderate to strong<br>…EQ-5D pain/discomfort → weak to moderate<br>…EQ-5D anxiety/depression → weak<br>**SF role participation** & …<br>…EQ-5D mobility → weak to moderate<br>…EQ-5D self-care → weak to moderate<br>…EQ-5D usual activities → moderate to strong<br>…EQ-5D pain/discomfort → weak<br>…EQ-5D anxiety/depression → moderate to strong<br>**SF social functioning** & …<br>…EQ-5D mobility → weak to moderate<br>…EQ-5D self-care → weak to moderate<br>…EQ-5D usual activities → moderate<br>…EQ-5D pain/discomfort → weak to moderate<br>…EQ-5D anxiety/depression → weak to moderate<br>**SF bodily pain** & …<br>…EQ-5D mobility → moderate<br>…EQ-5D self-care → moderate<br>…EQ-5D usual activities → moderate<br>…EQ-5D pain/discomfort → strong<br>…EQ-5D anxiety/depression → weak to moderate<br>**SF mental health** & …<br>…EQ-5D mobility → weak<br>…EQ-5D self-care → weak<br>…EQ-5D usual activities → weak to moderate<br>…EQ-5D pain/discomfort → weak to moderate<br>…EQ-5D anxiety/depression → strong<br>**SF vitality** & …<br>…EQ-5D mobility → weak to moderate<br>…EQ-5D self-care → weak to moderate<br>…EQ-5D usual activities → weak to moderate<br>…EQ-5D pain/discomfort → weak to moderate<br>…EQ-5D anxiety/depression → weak to moderate<br>**BBS** & …<br>…EQ-5D mobility → moderate to strong correlation

	…EQ-5D self-care → moderate<br><br>…EQ-5D usual activities → low to moderate<br><br>…EQ-5D pain/discomfort → low to moderate<br><br>…EQ-5D depression/anxiety → low or no association<br><br>**30s STS** & …<br><br>…EQ-5D mobility → moderate to strong<br><br>…EQ-5D self-care → low to moderate<br><br>…EQ-5D usual activities → low to moderate<br><br>…EQ-5D pain/discomfort → low to moderate<br><br>…EQ-5D depression/anxiety → low or no association<br><br>**4m-walk test** & …<br><br>…EQ-5D mobility → moderate to strong<br><br>…EQ-5D self-care → low to moderate<br><br>…EQ-5D usual activities → low to moderate<br><br>…EQ-5D pain/discomfort → low to moderate<br><br>…EQ-5D depression/anxiety → low or no association<br><br>**FESI** & …<br><br>…EQ-5D mobility → moderate to strong<br><br>…EQ-5D self-care → moderate<br><br>…EQ-5D usual activities → moderate to strong<br><br>…EQ-5D pain/discomfort → low to moderate<br><br>…EQ-5D depression/anxiety → moderate<br><br>*Responsiveness – Comparison with other instruments:*<br><br>**BBS** → moderate association of change scores<br><br>**30s STS** → weak association of change scores<br><br>**4m-walk test** → weak association of change scores<br><br>**FESI** → weak to moderate association of change scores
Brazier et al.[7]	*Known-groups validity* → *(H5)*<br><br>Age → H7<br><br>GP visit in previous 14d = yes → lower EQ-5D values<br><br>Outpatient attendance in previous 3 months = yes → lower EQ-5D values<br><br>Accident and Emergency department attendance in prev. 3 months = yes → lower EQ-5D values<br><br>Hospital inpatient stay in prev. 12 months = yes → lower EQ-5D values<br><br>Longstanding illness = yes → lower EQ-5D values<br><br>Higher disability severity (OPCS) is associated with lower EQ-5D values

Cheng et al.[8]	*Convergent validity:*<br><br>**EQ-VAS** → moderate to strong<br><br>**SPVU-5D** → moderate to strong<br><br>**SPVU-5D pain** & …<br><br>…EQ-5D mobility → weak to moderate<br><br>… EQ-5D self-care → weak to moderate<br><br>… EQ-5D usual activities → weak to moderate<br><br>… EQ-5D pain/disc. → strong<br><br>… EQ-5D anxiety/depression → weak to moderate<br><br>**SPVU-5D mobility** & …<br><br>… EQ-5D mobility → strong<br><br>… EQ-5D self-care → moderate<br><br>… EQ-5D usual activities → moderate to strong<br><br>… EQ-5D pain/discomfort → weak to mod.<br><br>… EQ-5D anxiety/depression → weak to moderate<br><br>**SPVU-5D mood** & …<br><br>… EQ-5D mobility → weak to moderate<br><br>… EQ-5D self-care → weak to moderate<br><br>… EQ-5D usual activities → weak to moderate<br><br>… EQ-5D pain/discomfort → weak to mod.<br><br>… EQ-5D anxiety/depression → mod. to strong<br><br>**SPVU-5D smell** & …<br><br>… EQ-5D mobility → weak to moderate<br><br>… EQ-5D self-care → weak or no assoc.<br><br>… EQ-5D usual activities → weak to moderate<br><br>… EQ-5D pain/discomfort → weak<br><br>… EQ-5D anxiety/depression → weak<br><br>**SPVU-5D social activities** & …<br><br>… EQ-5D mobility → moderate to strong<br><br>… EQ-5D self-care → weak to moderate<br><br>… EQ-5D usual activities → moderate to strong<br><br>… EQ-5D pain/disc. → weak to mod.<br><br>… EQ-5D anxiety/depression → weak to moderate<br><br>*Known-groups validity:*<br><br>**Age** (mean/median split) → low to mod. effect size (ES)

	**Duration of ulcer** (mean/median split) → mod. ES<br>**Healing status** (mean/median split) → mod. ES<br>**EQ-VAS** (bad-fair-good-excellent) → meaningful differences bw. groups with lower VAS values having lower EQ-5D values<br>*Responsiveness – Comparison bw. subgroups:*<br>Change scores for those with healed VLUs at different FU time points exceed the change scores for those who remained unhealed.<br>People with longer active ulcer duration (=proxy for ulcer severity) have lowest changes in EQ-5D index from BL to FU
Coast et al.[9]	*Convergent validity:*<br>EQ-5D mobility & Barthel index → moderate to strong<br>EQ-5D mobility & Barthel transfer → moderate to strong<br>EQ-5D mobility & Barthel mobility → strong<br>EQ-5D mobility & Barthel stairs → moderate to strong<br>EQ-5D self-care & Bartel index → moderate to strong<br>EQ-5D self-care & Bartel grooming → strong<br>EQ-5D self-care & Bartel toilet use → moderate to strong<br>EQ-5D self-care & Bartel feeding → moderate to strong<br>EQ-5D self-care & Bartel dressing/bathing → strong<br>**COOP-WONCA physical fitness** & …<br>… EQ-5D index → moderate to strong<br>…EQ-5D mobility → moderate to strong<br>…EQ-5D self-care → weak to moderate<br>…EQ-5D usual activities → weak to moderate<br>…EQ-5D pain/discomfort → weak to moderate<br>…EQ-5D anxiety/depression → weak<br>**COOP-WONCA feelings** & …<br>… EQ-5D index → weak to moderate<br>… EQ-5D mobility → weak to moderate<br>… EQ-5D usual activities → weak to moderate<br>… EQ-5D self-care → weak to moderate<br>… EQ-5D pain/discomfort → weak<br>… EQ-5D anxiety/depression → mod. to strong<br>**COOP-WONCA daily activities** & …<br>… EQ-5D index → moderate to strong<br>… EQ-5D mobility → moderate to strong

	… EQ-5D self-care → moderate to strong<br><br>… EQ-5D usual activities → strong<br><br>… EQ-5D pain/discomfort → weak to moderate<br><br>… EQ-5D anxiety/depression → weak to mod.<br><br>**COOP-WONCA social activities** & …<br><br>… EQ-5D index → moderate<br><br>… EQ-5D mobility → weak to moderate<br><br>… EQ-5D self-care → weak to moderate<br><br>… EQ-5D usual activities → moderate<br><br>… EQ-5D pain/discomfort → weak to moderate<br><br>… EQ-5D anxiety/depression → weak to moderate<br><br>**COOP-WONCA change in health** & …<br><br>… EQ-5D index → moderate<br><br>… EQ-5D mobility → moderate<br><br>… EQ-5D self-care → weak<br><br>… EQ-5D usual activities → weak to moderate<br><br>… EQ-5D pain/discomfort → weak<br><br>… EQ-5D anxiety/depression → weak to mod.<br><br>**COOP-WONCA overall health** & …<br><br>… EQ-5D index → strong<br><br>… EQ-5D mobility → moderate to strong<br><br>… EQ-5D self-care → weak to moderate<br><br>… EQ-5D usual activities → weak to mod.<br><br>… EQ-5D pain/discomfort → weak to moderate<br><br>… EQ-5D anxiety/depression → weak to mod.<br><br>**COOP-WONCA pain** & …<br><br>… EQ-5D index → moderate<br><br>… EQ-5D mobility → weak to moderate<br><br>… EQ-5D self-care → weak to moderate<br><br>… EQ-5D usual activities → weak to moderate<br><br>… EQ-5D pain/discomfort → strong<br><br>… EQ-5D anxiety/depression → weak<br><br>*Known-groups validity:*<br><br>**Age** & EQ-5D → H7<br><br>People with non-limiting or limiting **disability** have lower HrQoL than people with no disability.

	Responsiveness – Comparison bw. subgroups:<br><br>People admitted for elective total hip/knee replacement, who are relatively fit & healthy prior to surgery recover more quickly than those admitted with fractured neck of femur, who could be expected to be more frail prior to their treatment → clinically significant difference in change score<br><br>People with fractured femur neck recover more quickly than those admitted with stroke → clinically sign. difference in change score
Davis et al.[10]	*Convergent validity:*<br><br>EQ-5D & **ICECAP-O** → moderate<br><br>**PPA & …**<br><br>…EQ-5D mobility → weak or no assoc.<br><br>…EQ-5D Self-care → weak or no assoc.<br><br>…EQ-5D Usual activities → weak or no assoc.<br><br>…EQ-5D Pain/Discomfort → weak or no assoc.<br><br>…EQ-5D Anxiety/Depression → weak or no assoc.<br><br>…EQ-5D Index → weak or no assoc.<br><br>**SPPB** & …<br><br>…EQ-5D Mobility → weak to moderate<br><br>…EQ-5D Self-care → weak or no<br><br>…EQ-5D Usual activities → weak or no<br><br>…EQ-5D Pain/Discomfort → weak or no<br><br>…EQ-5D Anxiety/Depression → no<br><br>…EQ-5D Index → weak or no<br><br>**MMSE** & …<br><br>…EQ-5D Mobility → weak/no<br><br>…EQ-5D Self-care→ weak/no<br><br>…EQ-5D Usual activities→ weak/no<br><br>…EQ-5D Pain/Discomfort → weak/no<br><br>…EQ-5D Anxiety/Depression → weak/no<br><br>…EQ-5D Index → weak/no<br><br>**IADL** & …<br><br>…EQ-5D Mobility → weak to moderate<br><br>…EQ-5D Self-care → weak to moderate<br><br>…EQ-5D Usual activities → moderate to strong<br><br>…EQ-5D Pain/Discomfort → weak to moderate<br><br>…EQ-5D Anxiety/Depression → weak/no<br><br>…EQ-5D Index → weak to moderate

Davis et al.[11]	*Responsiveness – Comparison bw. subgroups*:<br><br>The change (decline) in HrQoL over time is larger in **fallers versus non-fallers.**
Easton et al.[12]	*Convergent validity:*<br><br>**DEMQOL-U index** & …<br><br>…EQ-5D Mobility → no/weak<br><br>…EQ-5D Self-Care → weak/moderate<br><br>…EQ-5D Usual activities → weak/moderate<br><br>…EQ-5D Pain/discomfort → weak/moderate<br><br>…EQ-5D Anxiety/Depression → weak/moderate<br><br>…EQ-5D Index → moderate<br><br>**DEMQOL-U positive emotion** & …<br><br>… EQ-5D Mobility → weak to moderate<br><br>… EQ-5D Self-Care → no/weak<br><br>… EQ-5D Usual activities → weak/moderate<br><br>… EQ-5D Pain/discomfort → weak<br><br>… EQ-5D Anxiety/Depression → weak/moderate<br><br>… EQ-5D Index → weak/moderate<br><br>**DEMQOL-U negative emotion** & …<br><br>… EQ-5D Index Mobility → no/weak<br><br>… EQ-5D Index Self-Care → no/weak<br><br>… EQ-5D Index Usual activities → weak/moderate<br><br>… EQ-5D Index Pain/discomfort → weak/moderate<br><br>… EQ-5D Index Anxiety/Depression → moderate/strong<br><br>… EQ-5D Index index → weak/moderate<br><br>**DEMQOL-U loneliness** & …<br><br>… EQ-5D Mobility → no/weak<br><br>… EQ-5D Self-Care & DEMQOL-U loneliness → no/weak<br><br>… EQ-5D Usual activities & DEMQOL-U loneliness → weak/moderate<br><br>… EQ-5D Pain/discomfort & DEMQOL-U loneliness → weak/no<br><br>… EQ-5D Anxiety/Depression & DEMQOL-U loneliness → weak/moderate<br><br>… EQ-5D Index & DEMQOL-U loneliness → weak<br><br>**DEMQOL-U cognition** & …<br><br>… EQ-5D Mobility→ weak/no<br><br>… EQ-5D Self-Care → weak/moderate<br><br>… EQ-5D Usual activities → weak/moderate

	… EQ-5D Pain/discomfort → weak/no<br>… EQ-5D Anxiety/Depression → weak<br>… EQ-5D Index → weak/no<br>**DEMQOL-U relationships** & …<br>… EQ-5D Mobility → weak/mod.<br>… EQ-5D Self-Care → weak/mod.<br>… EQ-5D Usual activities → weak/moderate<br>… EQ-5D Pain/discomfort → weak/no<br>… EQ-5D Anxiety/Depression → weak/no<br>… EQ-5D Index → weak/moderate<br>**Pas-Cog** (cognitive impairment) &…<br>… EQ-5D Mobility → weak<br>… EQ-5D Self-Care → weak/moderate<br>… EQ-5D Usual activities → weak/moderate<br>… EQ-5D Pain/discomfort → no/weak<br>… EQ-5D Anxiety/Depression → weak/no<br>… EQ-5D Index → weak/moderate<br>Mobility & **MBI** (physical function) & …<br>… EQ-5D mobility → moderate<br>… EQ-5D Self-Care → moderate to strong<br>… EQ-5D Usual activities → moderate<br>… EQ-5D Pain/discomfort → no/weak<br>… EQ-5D Anxiety/Depression → no/weak<br>… EQ-5D Index → moderate<br>**NPI-Q** (neuropsychiatric symptoms) & …<br>… EQ-5D mobility → weak/no<br>… EQ-5D Self-Care → weak/no<br>… EQ-5D Usual activities → weak/no<br>… EQ-5D Pain/discomfort → weak/no<br>… EQ-5D Anxiety/Depression → weak/no<br>… EQ-5D Index → weak/no<br>*Known-groups validity*:<br>People with no/mild **cognitive impairment** (CI) have higher HrQoL than those with moderate/severe CI<br>People with better **physical functioning** have better HrQoL than those with worse physical functioning
Frihagen et al.[13]	*Responsiveness – Comparison bw. subgroups*:

	The complications group has lower EQ-5D values at 4 and at 12 months than the non-complications group<br><br>The mean change bw. 4 and 12 months is higher in the complications group than in the non-complications group.
Griffiths et al.[14]	*Convergent validity:*<br><br>**QOL-AD** → moderate
Hazell et al. [15]	*Known-groups validity:*<br><br>People with likely obstructive airways disease have lower EQ-5D scores than people with unlikely obstructive airways disease.
Heiskanen et al. [16]	*Convergent validity:*<br><br>**15D** → strong<br><br>*Responsiveness – Comparison with other instruments*:<br><br>The proportions of changes stratified according to the MID values are similar between EQ-5D and 15D.
Holland et al.[17]	*Convergent validity:*<br><br>**AQOL** → moderate to strong<br><br>*Known-groups validity:*<br><br>**sex**: Women have lower scores than men<br><br>**age** → H7<br><br>**social class** → H8<br><br>people taking more **medications** report lower scores<br><br>people **living alone** report higher scores than those not living alone<br><br>according to the population (neither severe dementia, nor institutionalized no (or only weak) correlation with abbreviated **mental test score** assumed (H9)<br><br>*Responsiveness – Comparison with other instruments:*<br><br>**AQOL** → moderate to strong correlation of change scores
Jönsson et al. [18]	*Convergent validity*:<br><br>**MMSE** → weak/no<br><br>**QoL-AD** → moderate to strong<br><br>**VAS** → moderate to strong<br><br><br>**MMSE** & …<br><br>… EQ-5D Mobility → weak or no association<br><br>… EQ-5D Self-care → weak or no association<br><br>… EQ-5D Usual activities → weak or no association<br><br>… EQ-5D Pain/Discomfort → weak or no association<br><br>… EQ-5D Anxiety/Depression → weak or no association
Kaambwa et al.[19]	*Convergent validity*:

	Correlation bw. **subdimensions of the EQ-5D** and **subdimensions of the OPQOL-Brief/ASCOT** according to the generic hypotheses<br><br>**OPQoL-Brief** Summary Score → moderate to strong<br><br>**ASCOT** Summary Score → moderate to strong<br><br>*Known-groups validity*:<br><br>**Age** → H7<br><br>**Sex**: Male = higher scores<br><br>**Living alon**e = higher scores<br><br>**Education** → H8<br><br>Having higher self-reported **general health** = higher scores<br><br>having **informal care support** = higher scores
Karlawish et al. [20]	*Known-groups validity*:<br><br>People with higher EQ-5D scores have higher scores in…:<br><br>• function (**ADL/IADL** [Lawton-Brody scales])<br>• mood (**GDS-15**)<br>• generic health-related QoL (**SF-12** general health, MCS, & PCS)<br>• **QOL-AD** dimensions Memory, Life & Whole<br>**MMSE (Cognition)** → H9<br><br>**GDS Memory** → H9
Kim et al. [21]	*Known-groups validity*:<br><br>Men with higher LUTS severity have lower EQ-5D scores.
Kunz [22]	*Convergent validity*:<br><br>**Barthel** Index → moderate<br><br>**NOSGER** subscale **IADL** → weak to moderate<br><br>**MMSE** → weak or no association<br><br>*Known-groups validity*:<br><br>People with **dementia as only co-morbidity** have a higher HrQoL than those with additional comorbidities.<br><br>*Responsiveness – Comparison with other instruments*:<br><br>**Barthel** Index → moderate corr. of change scores<br><br>**NOSGER** subscale **IADL** → weak to moderate corr. of change scores<br><br>**MMSE** → weak or no corr. of change scores<br><br>*Responsiveness – Comparison bw. subgroups:*<br><br>People with **deterioration in health status** (CGI-I) have a higher effect size in EQ-5D change bw. BL & FU.
Lutomski et al.[23]	*Convergent validity*:<br><br>EQ-5D mobility & **Katz** assistance with walking → strong

	EQ-5D self-care & Katz bathing → strong<br><br>EQ-5D self-care & Katz dressing → strong<br><br>EQ-5D usual activities & Katz summary IADL score → strong<br><br>EQ-5D anxiety/depression & **Rand-36** mental health sub scale → moderate to strong<br><br>EQ-5D index & **Cantril's Self Anchoring Ladder** → moderate<br><br>*Known-groups validity*:<br><br>EQ-5D value is lower in people who are …:<br><br>• older (or H7!)<br>• widowed/single<br>• lower educated (or H8!)<br>• living alone<br>• women (especially in dimension anxiety/depression)<br>• multi-morbid
Malkin et al.[24]	*Convergent validity*:<br><br>Items of the filtered **Activity Inventory** for LVR patients & EQ-5D index:<br><br>Reading → weak to moderate<br><br>Mobility → weak to moderate<br><br>Vis Motor → weak to moderate<br><br>Vis info → weak to moderate<br><br>Goals → weak to moderate<br><br>*Responsiveness – Comparison with other instruments:*<br><br>**Visual ability** → weak to moderate association of change scores<br><br>*Responsiveness – Before and after intervention*:<br><br>Low vision care has a clinically important effect on HrQoL (EQ-5D).
Martin et al.[25]	*Convergent validity:*<br><br>**QOL-AD-NH** → moderate<br><br>**CDR** → weak or no association<br><br>**FAST** → weak to moderate<br><br>**CMAI** → weak or no association<br><br>*Responsiveness – Comparison with other instruments:*<br><br>**QOL-AD-NH** →moderate association of change scores<br><br>**CDR** → no or weak association of change scores<br><br>**FAST** → weak to moderate association of change scores<br><br>**CMAI** → no or weak association of change scores
Michalowsky et al.[26]	*Convergent validity:*<br><br>**QoL-AD** → moderate to strong<br><br>**General health status** (Proxy) → moderate to strong

	**Lawton IADL** → weak to moderate<br><br>**GDS** → weak to moderate<br><br>*Known-groups validity:*<br><br>Lower EQ-5D scores for people with:<br><br>• poorer/better **general health** status<br>• more severe **IADL problems**<br>• higher **functional impairment due to dementia** have lower EQ-5D scores.<br>• more severe **depression** have lower EQ-5D scores.
Naglie et al.[27]	*Convergent validity:*<br><br>**Global health** → moderate to strong<br><br>**MMSE** → weak or no association<br><br>**Katz ADL** → moderate to strong<br><br>**Lawton IADL** → weak to moderate<br><br>**GDS** → weak to moderate<br><br>**Comorbidities** → weak to moderate<br><br>**QWB** → moderate to strong<br><br>**HUI3** → strong<br><br>**EQ-VAS** → moderate/strong
Nikolova et al.[28]	*Convergent validity:*<br><br>**SF-6D** → strong<br><br>**SF physical functioning** & …<br><br>…EQ-5D mobility → moderate to strong<br><br>…EQ-5D self-care → moderate to strong<br><br>…EQ-5D usual activities → moderate to strong<br><br>…EQ-5D pain/discomfort → weak to moderate<br><br>…EQ-5D anxiety/depression → weak<br><br>**SF role participation/limitation** & …<br><br>…EQ-5D mobility → weak to moderate<br><br>…EQ-5D self-care → weak to moderate<br><br>…EQ-5D usual activities → moderate to strong<br><br>…EQ-5D pain/discomfort → weak<br><br>…EQ-5D anxiety/depression → moderate to strong<br><br>**SF social functioning** & …<br><br>…EQ-5D mobility → weak to moderate<br><br>…EQ-5D self-care → weak to moderate<br><br>…EQ-5D usual activities → moderate

	…EQ-5D pain/discomfort → weak to moderate<br>…EQ-5D anxiety/depression → weak to moderate<br>**SF bodily pain** & …<br>…EQ-5D mobility → moderate<br>…EQ-5D self-care → moderate<br>…EQ-5D usual activities → moderate<br>…EQ-5D pain/discomfort → strong<br>…EQ-5D anxiety/depression → weak to moderate<br>**SF mental health** & …<br>…EQ-5D mobility → weak<br>…EQ-5D self-care → weak<br>…EQ-5D usual activities → weak to moderate<br>…EQ-5D pain/discomfort → weak to moderate<br>…EQ-5D anxiety/depression → strong<br>**SF vitality** & …<br>…EQ-5D mobility → weak to moderate<br>…EQ-5D self-care → weak to moderate<br>…EQ-5D usual activities → weak to moderate<br>…EQ-5D pain/discomfort → weak to moderate<br>…EQ-5D anxiety/depression → weak to moderate
Olerud et al.[29]	*Responsiveness – Comparison with other instruments*:<br>**DASH** → ≥ moderate correlation of change scores<br>*Responsiveness – Comparison btw. subgroups:*<br>The EQ-5D score is able to significantly discriminate bw. the dichotomized outcomes (AUC ≥ 0.7) .<br>Improvement in EQ-5D scores bw. 4 and 12 months in patients whose disease status (according to the EC) improved, but further deterioration in people whose disease status deteriorated.<br>*Responsiveness – Before & after intervention*:<br>Deterioration of HrQoL at 4 months compared with before fracture.
Orgeta et al.[30]	*Convergent validity:*<br>**CSDD** → weak to moderate<br>**RAID** → weak to moderate<br>**BADLS** → moderate
Parsons et al.[31]	*Convergent validity:*<br>**OHS** → moderate to strong<br>**ICECAP-O** → moderate<br>*Responsiveness – Comparison btw. subgroups:*

	The EQ-5D is able to predict death or revision (AUC ≥ 0.7)<br><br>*Responsiveness – Before & after intervention*:<br><br>Clinically important deterioration in EQ-5D bw. baseline (pre-fracture) & FU, but improvement in EQ-5D from 4 weeks to 4 months FU.
Pérez-Ros et al.[32]<br><br>(EQ-5D-3L for assessing…)	*Convergent validity:*<br><br>**Tinetti Index** → weak to moderate<br><br>**Barthel index** → moderate to strong<br><br>**Lawton index** → moderate to strong<br><br>**VAS pain** → weak to moderate<br><br>**GDS** → weak<br><br>Tinetti Index & EQ-5D mobility → moderate to strong<br><br>Barthel index & EQ-5D self-care → moderate to strong<br><br>Lawton index & EQ-5D usual activities → moderate to strong<br><br>VAS pain & EQ-5D pain → strong<br><br>GDS & EQ-5D anxiety → moderate to strong
Pérez-Ros et al.[33]	Convergent validity:<br><br>**EQ-VAS** → moderate to strong<br><br>Tinetti Index & EQ-5D mobility → moderate to strong<br><br>Barthel index & EQ-5D self-care → moderate to strong<br><br>Lawton index & EQ-5D usual activities → moderate to strong<br><br>VAS pain & EQ-5D pain → strong<br><br>GDS & EQ-5D anxiety → moderate to strong
Ratcliffe et al.[34]	*Convergent validity*:<br><br>**MMSE** → weak<br><br>**CSDD** → weak to moderate<br><br>**MBI** → moderate to strong<br><br>**PainAd** → weak to moderate<br><br>*Known-group validity*:<br><br>Lower EQ-5D scores in people with…:<br><br>• higher pain levels<br>• higher depression levels<br>• lower functioning levels
Sanchez-Arenas et al.[35]	*Convergent validity*:<br><br>**ADL** → moderate to strong<br><br>**IADL** → weak to moderate<br><br>**MMSE** → weak/no

	**SF-36 physical function** → moderate to strong<br><br>**SF-36 physical role** → moderate to strong<br><br>**SF-36 bodily pain** → moderate to strong<br><br>**SF-36 general health** → moderate to strong<br><br>**SF-36 vitality** → moderate to strong<br><br>**SF-36 social functioning** → moderate to strong<br><br>**SF-36 emotional role** → at least weak to moderate<br><br>**SF-36 mental health**→ moderate to strong<br><br>**Charlson comorbidity index** → moderate to strong
Tidermark et al.[36]	*Convergent validity*:<br><br>**SF-36** → strong<br><br>*Responsiveness – Comparison with other instruments*:<br><br>**SF-36** global score→ strong<br><br>**SF-36 physical function**→ moderate to strong<br><br>**SF-36 physical role** → moderate to strong<br><br>**SF-36 bodily pain** → moderate to strong<br><br>**SF-36 general health** → moderate to strong<br><br>**SF-36 vitality** → moderate to strong<br><br>**SF-36 social functioning** → moderate to strong<br><br>**SF-36 emotional role** → weak to moderate<br><br>**SF-36 mental health**→ moderate to strong<br><br>*Responsiveness – Comparison bw. subgroups*:<br><br>People with a "less good outcome" (pain >1 on the modified pain score and/or need for walking aids more than just one stick) score sign. lower in the EQ-5D sub-dimensions than people with a "good outcome"
Tidermark et al.[37]	*Responsiveness – Comparison with other instruments*:<br><br>**NHP** total score → strong<br><br>EQ-5D mobility & NHP phys. mobility → strong<br><br>EQ-5D pain/discomfort & NHP pain→ strong<br><br>EQ-5D anxiety/depression & NHP emotional reaction → strong<br><br>*Responsiveness – Comparison bw. subgroups*:<br><br>The mean change in EQ-5D scores bw. BL & 6-month FU is higher (negative direction, moderate to high SRM) for participants with displaced fracture than for participants with undisplaced fractures who remain relatively unchanged<br><br>The EQ-5D is able to discriminate bw. displaced and undisplaced fractures
van Leeuwen et al.[38]	*Convergent validity*:<br><br>**ICECAP-O** → moderate

	**ASCOT** → moderate<br><br>**Health GRS** → moderate to strong<br><br>**Katz ADL Index** → moderate to strong<br><br>**SF-12 PCS** → moderate to strong<br><br>**SF-12 MCS** → weak to moderate<br><br>**QoL GRS** → moderate<br><br>**Pearlin Mastery Scale** → weak to moderate<br><br>**CCCQ** → weak or no association<br><br>*Responsiveness – Comparison with other instruments*:<br><br>**ICECAP-O** → moderate<br><br>**ASCOT** → moderate<br><br>**Health GRS** → moderate to strong<br><br>**Katz ADL Index** → moderate to strong<br><br>**SF-12 PCS** → moderate to strong<br><br>**SF-12 MCS** → weak to moderate<br><br>**QoL GRS** → moderate<br><br>**Pearlin Mastery Scale** → weak to moderate<br><br>**CCCQ** → weak or no association
Walters et al.[39]	*Convergent validity*:<br><br>**SF-36 physical function** → moderate to strong<br><br>**SF-36 physical role** → moderate to strong<br><br>**SF-36 bodily pain** → moderate to strong<br><br>**SF-36 general health** → moderate to strong<br><br>**SF-36 vitality** → moderate to strong<br><br>**SF-36 social functioning** → moderate to strong<br><br>**SF-36 emotional role** → weak to moderate<br><br>**SF-36 mental health**→ moderate to strong<br><br>*Known-group validity*:<br><br>**Age** → H7<br><br>Lower EQ-5D scores in people …:<br><br>• with **larger leg ulcers**<br>• who have to **walk with an aid** or are chair or bed bound compared to those who are able to walk freely<br>• with longer ulcer duration<br>*Responsiveness – Comparison bw. subgroups*:<br><br>Higher deterioration of HrQoL (EQ-5D) in people whose ulcer had not healed at 3-month FU.

	EQ-5D scores improve more (or deteriorate less) in people whose perceived health change at 3M-FU is “better” or “same” than those who perceive it as “worse”<br><br>The change scores for people having a non-healed/recurred ulcer and those whose initial ulcer healed and stayed healed differ at 1 year FU.

Table S3 *Summary of findings – EQ-5D-3L – subdimensions.*

Measurement property	Summary	Overall rating
**Construct validity**		
**Convergent validity**		**+ (91%)**
EQ-5D index and comparison instruments' subdimensions		
HrQoL instruments	SF-36 [35, 39] (16/16), COOP-WONCA [9] (7/7)	+ (100%)
QoL instruments	ASCOT [40] (8/8), OPQoL-Brief [40] (13/13)	+ (100%)
Other instruments	Activity Inventory [24] (5/5)	+ (100%)
EQ-5D subdimensions and comparison instruments' summary scores		
QoL instruments	OPQoL-Brief [40] (5/5), ASCOT [40] (5/5)	+ (100%)
(I)ADL	Barthel [32, 33, 41] (7/7), Lawton & Brody [10, 32, 33] (4/7), Katz [23] (1/1)	+ (80%)
Cognitive status	MMSE‡ [10, 18] (10/10)	+ (100%)
Depression/anxiety	Rand-36 Mental Health [23] (1/1), EQ-5D anxiety/depression & GDS [32, 33] (1/2)	± (67%)
Other	PPA [10] (5/5), SPPB [10] (5/5), Tinetti [32, 33] (1/2), EQ-5D pain & VAS Pain [32, 33] (1/2), Age [3] (5/5)	+ (89%)
EQ-5D subdimensions and comparison instruments' sub dimensions		
HrQoL instruments	COOP-WONCA [9] (34/45)	+ (76%)
QoL instruments	OPQoL-Brief [40] (63/65), ASCOT [40] (38/40)	+ (96%)
ADL	Katz [23] (2/3)	± (67%)
**Responsiveness**		
**Comparison with other instruments**		± (36%)
EQ-5D index and comparison instruments' subdimensions		
HrQoL instruments	NHP [37] (0/3), SF-36 (4/8) [36]	± (36%)

‡ *no relevant difference between groups hypothesized*

Abbreviations: (x/y), x of y hypotheses supported; ADL, Activities of Daily Living; ASCOT, Adult Social Care Outcomes Toolkit; COOP/WONCA, Darmouth COOP Functional Health Assessment Charts/WONCA; EQ-5D, EuroQol five-dimensional questionnaire; GDS, Geriatric Depression Scale; HrQoL, health-related quality of life; MMSE, Mini-Mental State Examination; NHP, Nottingham Health Profile; OPQoL-Brief, Older People's Quality of Life questionnaire brief version; PPA, Physiological Profit Assessment; QoL, quality of life; SF-36, 36-Item Short-Form Health Survey; SPPB, Short Physical Performance Battery, VAS Pain, Visual Analog Scale for Pain.

Table S4 *Summary of findings – EQ-5D-5L – subdimensions.*

Measurement property	Summary	Overall rating
**Construct validity**		
**Convergent validity**		**+ (90%)**
<u>EQ-5D index and comparison instruments' subdimensions</u>		
QoL instruments	DEMQOL-U [12] (5/5)	+ (100%)
<u>EQ-5D subdimensions and comparison instruments' summary scores</u>		
QoL instruments	DEMQOL-U [12] (5/5)	+ (100%)
ADL	Barthel [41] (5/5), MBI [12] (5/5)	+ (100%)
Cognitive status	PAS-Cog* [12] (1/5)	– (20%)
Other instruments	NPI-Q [12] (5/5), 30s STS [6] (5/5), 4m walk test [6] (5/5), FES-I [6] (5/5), BBS [6] (5/5)	+ (100%)
<u>EQ-5D sub dimensions and comparison instruments' subdimensions</u>		
HrQoL instruments	SF-6D [6] (27/30), SF-6D [28] (28/30)	+ (92%)
QoL instruments	SPVU-5D [8] (23/25), DEMQOL-U [12] (22/25)	+ (90%)

‡ *no relevant difference between groups hypothesized*

* *results in the opposite of the hypothesized direction (H9)*

<u>*Abbreviations*</u>*; ADL, Activities of Daily Living; BBS, Berg Balance Scale; DEMQOL, Dementia Quality of Life; EQ-5D, EuroQol five-dimensional questionnaire; FES-I, Falls Efficacy Scale International; HrQoL, health-related quality of life; MBI, Modified Barthel Index; NPI-Q, Neuropsychiatric Inventory-Questionnaire; PAS-Cog, Psychogeriatric Assessment Scales-Cognitive Impairment Scale; QoL, quality of life; SF-6D, six-dimensional Short-Form Health Survey;30s STS, 30s sit-to-stand test; SPVU-5D, five-dimensional Sheffield-Preference-based Venous Ulcer questionnaire.*

Table S5 PRISMA checklist

Section/topic	#	Checklist item	Reported on page #
**TITLE**			
Title	1	Identify the report as a systematic review, meta-analysis, or both.	✓ (Title page)
**ABSTRACT**			
Structured summary	2	Provide a structured summary including, as applicable: background; objectives; data sources; study eligibility criteria, participants, and interventions; study appraisal and synthesis methods; results; limitations; conclusions and implications of key findings; systematic review registration number.	✓
**INTRODUCTION**			
Rationale	3	Describe the rationale for the review in the context of what is already known.	✓
Objectives	4	Provide an explicit statement of questions being addressed with reference to participants, interventions, comparisons, outcomes, and study design (PICOS).	✓
**METHODS**			
Protocol and registration	5	Indicate if a review protocol exists, if and where it can be accessed (e.g., Web address), and, if available, provide registration information including registration number.	✓
Eligibility criteria	6	Specify study characteristics (e.g., PICOS, length of follow-up) and report characteristics (e.g., years considered, language, publication status) used as criteria for eligibility, giving rationale.	✓
Information sources	7	Describe all information sources (e.g., databases with dates of coverage, contact with study authors to identify additional studies) in the search and date last searched.	✓
Search	8	Present full electronic search strategy for at least one database, including any limits used, such that it could be repeated.	✓ (Table S1, Supplement 1)
Study selection	9	State the process for selecting studies (i.e., screening, eligibility, included in systematic review, and, if applicable, included in the meta-analysis).	✓
Data collection process	10	Describe method of data extraction from reports (e.g., piloted forms, independently, in duplicate) and any processes for obtaining and confirming data from investigators.	✓
Data items	11	List and define all variables for which data were sought (e.g., PICOS, funding sources) and any assumptions and simplifications made.	N/A
Risk of bias in individual studies	12	Describe methods used for assessing risk of bias of individual studies (including specification of whether this was done at the study or outcome level), and how this information is to be used in any data synthesis.	✓
Summary measures	13	State the principal summary measures (e.g., risk ratio, difference in means).	✓
Synthesis of results	14	Describe the methods of handling data and combining results of studies, if done, including measures of consistency (e.g., I^2) for each meta-analysis.	✓

Section/topic	#	Checklist item	Reported on page #
Risk of bias across studies	15	Specify any assessment of risk of bias that may affect the cumulative evidence (e.g., publication bias, selective reporting within studies).	N/A
Additional analyses	16	Describe methods of additional analyses (e.g., sensitivity or subgroup analyses, meta-regression), if done, indicating which were pre-specified.	N/A
**RESULTS**			
Study selection	17	Give numbers of studies screened, assessed for eligibility, and included in the review, with reasons for exclusions at each stage, ideally with a flow diagram.	✓ (Figure 1)
Study characteristics	18	For each study, present characteristics for which data were extracted (e.g., study size, PICOS, follow-up period) and provide the citations.	✓ (Table 2)
Risk of bias within studies	19	Present data on risk of bias of each study and, if available, any outcome level assessment (see item 12).	✓ (Supplement)
Results of individual studies	20	For all outcomes considered (benefits or harms), present, for each study: (a) simple summary data for each intervention group (b) effect estimates and confidence intervals, ideally with a forest plot.	N/A
Synthesis of results	21	Present results of each meta-analysis done, including confidence intervals and measures of consistency.	N/A
Risk of bias across studies	22	Present results of any assessment of risk of bias across studies (see Item 15).	✓
Additional analysis	23	Give results of additional analyses, if done (e.g., sensitivity or subgroup analyses, meta-regression [see Item 16]).	N/A
**DISCUSSION**			
Summary of evidence	24	Summarize the main findings including the strength of evidence for each main outcome; consider their relevance to key groups (e.g., healthcare providers, users, and policy makers).	✓
Limitations	25	Discuss limitations at study and outcome level (e.g., risk of bias), and at review-level (e.g., incomplete retrieval of identified research, reporting bias).	✓
Conclusions	26	Provide a general interpretation of the results in the context of other evidence, and implications for future research.	✓
**FUNDING**			
Funding	27	Describe sources of funding for the systematic review and other support (e.g., supply of data); role of funders for the systematic review.	✓ (Title page)

From: Moher D, Liberati A, Tetzlaff J, Altman DG, The PRISMA Group (2009). Preferred Reporting Items for Systematic Reviews and Meta-Analyses: The PRISMA Statement. PLoS Med 6(7): e1000097. doi:10.1371/journal.pmed1000097

For more information, visit: **www.prisma-statement.org**.

References

1. Terwee, C. B., Jansma, E. P., Riphagen, I. I., & de Vet, H. C. W. (2009). Development of a methodological PubMed search filter for finding studies on measurement properties of measurement instruments. *Quality of Life Research*, 18(8), 1115-1123. https://doi.org/10.1007/s11136-009-9528-5
2. Aguirre, E., Kang, S., Hoare, Z., Edwards, R. T., & Orrell, M. (2016). How does the EQ-5D perform when measuring quality of life in dementia against two other dementia-specific outcome measures? *Quality of Life Research*, 25(1), 45-49. https://doi.org/10.1007/s11136-015-1065-9
3. Ankri, J., Beaufils, B., Novella, J. L., Morrone, I., Guillemin, F., Jolly, D., Ploton, L., & Blanchard, F. (2003). Use of the EQ-5D among patients suffering from dementia. *Journal of clinical epidemiology.*, 56(11), 1055-1063. https://doi.org/10.1016/s0895-4356(03)00175-6
4. Barton, G. R., Sach, T. H., Avery, A. J., Jenkinson, C., Doherty, M., Whynes, D. K., & Muir, K. R. (2008). A comparison of the performance of the EQ-5D and SF-6D for individuals aged >or= 45 years. *Health Economics*, 17(7), 815-832. https://doi.org/10.1002/hec.1298
5. Bhadhuri, A., Kind, P., Salari, P., Jungo, K. T., Boland, B., Byrne, S., Hossmann, S., Dalleur, O., Knol, W., Moutzouri, E., O'Mahony, D., Murphy, K. D., Wisselink, L., Rodondi, N., & Schwenkglenks, M. (2020). Measurement properties of EQ-5D-3L and EQ-5D-5L in recording self-reported health status in older patients with substantial multimorbidity and polypharmacy. *Health and Quality of Life Outcomes*, 18(1), 317. https://doi.org/10.1186/s12955-020-01564-0
6. Bjerk, M., Brovold, T., Davis, J. C., & Bergland, A. (2019). Evaluating a falls prevention intervention in older home care recipients: a comparison of SF-6D and EQ-5D. *Quality of Life Research*, 28(12), 3187-3195. https://doi.org/10.1007/s11136-019-02258-x
7. Brazier, J. E., Walters, S. J., Nicholl, J. P., & Kohler, B. (1996). Using the SF-36 and Euroqol on an elderly population. *Quality of Life Research*, 5(2), 195-204. https://doi.org/10.1007/bf00434741
8. Cheng, Q., Kularatna, S., Lee, X. J., Graves, N., & Pacella, R. E. (2019). Comparison of EQ-5D-5L and SPVU-5D for measuring quality of life in patients with venous leg ulcers in an Australian setting. *Quality of Life Research*, 28(7), 1903-1911. https://doi.org/10.1007/s11136-019-02128-6
9. Coast, J., Peters, T. J., Richards, S. H., & Gunnell, D. J. (1998). Use of the EuroQoL among elderly acute care patients. *Quality of Life Research*, 7(1), 1-10. https://doi.org/10.1023/a:1008857203434
10. Davis, J. C., Bryan, S., McLeod, R., Rogers, J., Khan, K., & Liu-Ambrose, T. (2012). Exploration of the association between quality of life, assessed by the EQ-5D and ICECAP-O, and falls risk, cognitive function and daily function, in older adults with mobility impairments. *BMC Geriatrics*, 12, 65. https://doi.org/10.1186/1471-2318-12-65
11. Davis, J. C., Best, J. R., Dian, L., Khan, K. M., Hsu, C. L., Chan, W., Cheung, W., & Liu-Ambrose, T. (2017). Are the EQ-5D-3L and the ICECAP-O responsive among older adults with impaired mobility? Evidence from the Vancouver Falls Prevention Cohort Study. *Quality of Life Research*, 26(3), 737-747. https://doi.org/10.1007/s11136-016-1487-z
12. Easton, T., Milte, R., Crotty, M., & Ratcliffe, J. (2018). An empirical comparison of the measurement properties of the EQ-5D-5L, DEMQOL-U and DEMQOL-Proxy-U for older people in residential care. *Quality of Life Research*, 27(5), 1283-1294. https://doi.org/10.1007/s11136-017-1777-0
13. Frihagen, F., Grotle, M., Madsen, J. E., Wyller, T. B., Mowinckel, P., & Nordsletten, L. (2008). Outcome after femoral neck fractures: A comparison of Harris Hip Score, Eq-5d and Barthel Index. *Injury-International Journal of the Care of the Injured*, 39(10), 1147-1156. https://doi.org/10.1016/j.injury.2008.03.027
14. Griffiths, A. W., Smith, S. J., Martin, A., Meads, D., Kelley, R., & Surr, C. A. (2020). Exploring self-report and proxy-report quality-of-life measures for people living with dementia in care homes. *Quality of Life Research*, 29(2), 463-472. https://doi.org/10.1007/s11136-019-02333-3
15. Hazell, M., Frank, T., & Frank, P. (2003). Health related quality of life in individuals with asthma related symptoms. *Respiratory Medicine*, 97(11), 1211-1218. https://doi.org/https://doi.org/10.1016/S0954-6111(03)00249-X
16. Heiskanen, J., Tolppanen, A.-M., Roine, R. P., Hartikainen, J., Hippeläinen, M., Miettinen, H., & Martikainen, J. (2016). Comparison of EQ-5D and 15D instruments for assessing the health-related quality of life in cardiac surgery patients. *European Heart Journal - Quality of Care and Clinical Outcomes*, 2(3), 193-200. https://doi.org/10.1093/ehjqcco/qcw002
17. Holland, R., Smith, R. D., Harvey, I., Swift, L., & Lenaghan, E. (2004). Assessing quality of life in the elderly: a direct comparison of the EQ-5D and AQoL. *Health Economics*, 13(8), 793-805. https://doi.org/10.1002/hec.858
18. Jönsson, L., Andreasen, N., Kilander, L., Soininen, H., Waldemar, G., Nygaard, H., Winblad, B., Jönhagen, M. E., Hallikainen, M., & Wimo, A. (2006). Patient- and Proxy-Reported Utility in Alzheimer Disease Using the EuroQoL. *Alzheimer Disease and Associated Disorders*, 20(1), 49-55. https://doi.org/10.1097/01.wad.0000201851.52707.c9
19. Kaambwa, B., Gill, L., McCaffrey, N., Lancsar, E., Cameron, I. D., Crotty, M., Gray, L., & Ratcliffe, J. (2015). An empirical comparison of the OPQoL-Brief, EQ-5D-3 L and ASCOT in a community dwelling population of older people. *Health and Quality of Life Outcomes*, 13, 164. https://doi.org/10.1186/s12955-015-0357-7
20. Karlawish, J. H., Zbrozek, A., Kinosian, B., Gregory, A., Ferguson, A., & Glick, H. A. (2008). Preference-based quality of life in patients with Alzheimer's disease. *Alzheimers Dement*, 4(3), 193-202. https://doi.org/10.1016/j.jalz.2007.11.019
21. Kim, S.-K., Kim, K.-H., Kim, S.-H., Yoo, S.-J., & Jeong, Y.-W. (2019). Health-related quality of life in adult males with lower urinary tract symptoms. *Quality of Life Research*, 28(9), 2419-2428. https://doi.org/10.1007/s11136-019-02205-w
22. Kunz, S. (2010). Psychometric properties of the EQ-5D in a study of people with mild to moderate dementia. *Quality of Life Research*, 19(3), 425-434. https://doi.org/10.1007/s11136-010-9600-1

23. Lutomski, J. E., Krabbe, P. F., Bleijenberg, N., Blom, J., Kempen, G. I., MacNeil-Vroomen, J., Muntinga, M. E., Steyerburg, E., Olde-Rikkert, M. G., & Melis, R. J. (2017). Measurement properties of the EQ-5D across four major geriatric conditions: Findings from TOPICS-MDS. *Health and Quality of Life Outcomes*, 15(1), 45. https://doi.org/10.1186/s12955-017-0616-x
24. Malkin, A. G., Goldstein, J. E., Perlmutter, M. S., & Massof, R. W. (2013). Responsiveness of the EQ-5D to the effects of low vision rehabilitation. *Optometry and vision science : official publication of the American Academy of Optometry*, 90(8), 799-805. https://doi.org/10.1097/opx.0000000000000005
25. Martin, A., Meads, D., Griffiths, A. W., & Surr, C. A. (2019). How Should We Capture Health State Utility in Dementia? Comparisons of DEMQOL-Proxy-U and of Self- and Proxy-Completed EQ-5D-5L. *Value in health*, 22(12), 1417-1426. https://doi.org/10.1016/j.jval.2019.07.002
26. Michalowsky, B., Xie, F., Kohlmann, T., Gräske, J., Wübbeler, M., Thyrian, J. R., & Hoffmann, W. (2020). Acceptability and Validity of the EQ-5D in Patients Living With Dementia. *Value in Health*, 23(6), 760-767. https://doi.org/10.1016/j.jval.2020.01.022
27. Naglie, G., Tomlinson, G., Tansey, C., Irvine, J., Ritvo, P., Black, S. E., Freedman, M., Silberfeld, M., & Krahn, M. (2006). Utility-based Quality of Life measures in Alzheimer's disease. *Quality of Life Research*, 15(4), 631-643. https://doi.org/10.1007/s11136-005-4364-8
28. Nikolova, S., Hulme, C., West, R., Pendleton, N., Heaven, A., Bower, P., Humphrey, S., Farrin, A., Cundill, B., Hawkins, R., & Clegg, A. (2020). Normative Estimates and Agreement Between 2 Measures of Health-Related Quality of Life in Older People With Frailty: Findings From the Community Ageing Research 75+ Cohort. *Value Health*, 23(8), 1056-1062. https://doi.org/10.1016/j.jval.2020.04.1830
29. Olerud, P., Tidermark, J., Ponzer, S., Ahrengart, L., & Bergström, G. (2011). Responsiveness of the EQ-5D in patients with proximal humeral fractures. *Journal of Shoulder and Elbow Surgery*, 20(8), 1200-1206. https://doi.org/10.1016/j.jse.2011.06.010
30. Orgeta, V., Edwards, R. T., Hounsome, B., Orrell, M., & Woods, B. (2015). The use of the EQ-5D as a measure of health-related quality of life in people with dementia and their carers. *Quality of Life Research*, 24(2), 315-324. https://doi.org/10.1007/s11136-014-0770-0
31. Parsons, N., Griffin, X. L., Achten, J., & Costa, M. L. (2014). Outcome assessment after hip fracture: is EQ-5D the answer? *Bone Joint Res*, 3(3), 69-75. https://doi.org/10.1302/2046-3758.33.2000250
32. Pérez-Ros, P., & Martínez-Arnau, F. M. (2020). EQ-5D-3L for Assessing Quality of Life in Older Nursing Home Residents with Cognitive Impairment. *Life (Basel)*, 10(7). https://doi.org/10.3390/life10070100
33. Pérez-Ros, P., Vila-Candel, R., Martin-Utrilla, S., & Martínez-Arnau, F. M. (2020). Health-Related Quality of Life in Community-Dwelling Older People with Cognitive Impairment: EQ-5D-3L Measurement Properties. *J Alzheimers Dis*, 77(4), 1523-1532. https://doi.org/10.3233/jad-200806
34. Ratcliffe, J., Flint, T., Easton, T., Killington, M., Cameron, I., Davies, O., Whitehead, C., Kurrle, S., Miller, M., Liu, E., & Crotty, M. (2017). An Empirical Comparison of the EQ-5D-5L, DEMQOL-U and DEMQOL-Proxy-U in a Post-Hospitalisation Population of Frail Older People Living in Residential Aged Care. *Applied Health Economics and Health Policy*, 15(3), 399-412. https://doi.org/10.1007/s40258-016-0293-7
35. Sanchez-Arenas, R., Vargas-Alarcon, G., Sanchez-Garcia, S., Garcia-Peña, C., Gutierrez-Gutierrez, L., Grijalva, I., Garcia-Dominguez, A., & Juárez-Cedillo, T. (2014). Value of EQ-5D in Mexican city older population with and without dementia (SADEM study). *International Journal of Geriatric Psychiatry*, 29(5), 478-488. https://doi.org/10.1002/gps.4030
36. Tidermark, J., Bergström, G., Svensson, O., Törnkvist, H., & Ponzer, S. (2003). Responsiveness of the EuroQol (EQ 5-D) and the SF-36 in elderly patients with displaced femoral neck fractures. *Quality of Life Research*, 12(8), 1069-1079. https://doi.org/10.1023/a:1026193812514
37. Tidermark, J., & Bergström, G. (2007). Responsiveness of the EuroQol (EQ-5D) and the Nottingham Health Profile (NHP) in elderly patients with femoral neck fractures. *Quality of Life Research*, 16(2), 321-330. https://doi.org/10.1007/s11136-006-9004-4
38. van Leeuwen, K. M., Bosmans, J. E., Jansen, A. P., Hoogendijk, E. O., van Tulder, M. W., van der Horst, H. E., & Ostelo, R. W. (2015). Comparing measurement properties of the EQ-5D-3L, ICECAP-O, and ASCOT in frail older adults. *Value in Health*, 18(1), 35-43. https://doi.org/10.1016/j.jval.2014.09.006
39. Walters, S. J., Morrell, C. J., & Dixon, S. (1999). Measuring health-related quality of life in patients with venous leg ulcers. *Quality of Life Research*, 8(4), 327-336. https://doi.org/10.1023/a:1008992006845
40. Kaambwa, B., Gill, L., McCaffrey, N., Lancsar, E., Cameron, I. D., Crotty, M., Gray, L., & Ratcliffe, J. (2015). An empirical comparison of the OPQoL-Brief, EQ-5D-3 L and ASCOT in a community dwelling population of older people. *Health and Quality of Life Outcomes*, 13(1), 164. https://doi.org/10.1186/s12955-015-0357-7
41. Bhadhuri, A., Kind, P., Salari, P., Jungo, K. T., Boland, B., Byrne, S., Hossmann, S., Dalleur, O., Knol, W., Moutzouri, E., O'Mahony, D., Murphy, K. D., Wisselink, L., Rodondi, N., & Schwenkglenks, M. (2020). Measurement properties of EQ-5D-3L and EQ-5D-5L in recording self-reported health status in older patients with substantial multimorbidity and polypharmacy. *Health Qual Life Outcomes*, 18(1), 317. https://doi.org/10.1186/s12955-020-01564-0
